# Phosphorus and Zinc Are Strongly Associated with Belowground Fungal Communities in Wheat Field under Long-Term Fertilization

**DOI:** 10.1128/spectrum.00110-22

**Published:** 2022-03-10

**Authors:** Di Wu, Yuying Ma, Teng Yang, Guifeng Gao, Daozhong Wang, Xisheng Guo, Haiyan Chu

**Affiliations:** a State Key Laboratory of Soil and Sustainable Agriculture, Institute of Soil Science, Chinese Academy of Sciences, Nanjing, China; b University of Chinese Academy of Sciences, Beijing, China; c Key Laboratory of Nutrient Cycling and Resources Environment of Anhui Province, Soil and Fertilizer Research Institute, Anhui Academy of Agricultural Sciences, Hefei, China; University of Minnesota

**Keywords:** long-term fertilization, fungal community, wheat field, habitats, phosphorus and zinc

## Abstract

Belowground fungi are closely related to crop growth, and agricultural fertilization is widely known to affect soil fungal communities. Yet it remains unclear whether fungal communities in differing belowground habitats—root endosphere, rhizosphere soil, and bulk soil—respond differently to long-term fertilization. Here we investigated the variation in fungal communities of root endosphere, rhizosphere soil, and bulk soil under 35 years of fertilization in wheat fields. Specifically, the fertilization regimes were applied as five treatments: soils receiving NPK fertilizer, NPK and cow manure (NPK+CM), NPK and pig manure (NPK+PM), NPK and wheat straw (NPK+WS), and no fertilizer (Control). Long-term fertilization significantly impacted fungal community composition in all three habitats, and these effects were stronger in the rhizosphere and bulk soils than root endosphere. Mantel test results showed that fungal community composition was significantly correlated with phosphorus and zinc contents. Further, fungal alpha diversity was lowest in the NPK+PM treatment and was negatively correlated with both phosphorus and zinc contents. Moreover, NPK+PM treatment had the lowest complexity of fungal co-occurrence network, and in general network complexity was significantly negatively correlated with the zinc and phosphorus contents. Taken together, these results suggest that long-term fertilization can impact fungal communities not only in soils but in root endosphere, and this is strongly associated with the contents of phosphorus and zinc there, a finding important for guiding fertilization management practices and supporting sustainable agriculture.

**IMPORTANCE** Fungi, an essential component in nutrient cycling and plant growth, are highly sensitive to fertilization. However, there are limited studies on fungi in root endosphere under long-term fertilization management. Our research extended the study on the endophytic fungal community of crop roots under agricultural management and found that its responses were similar to the communities in soil habitats. In addition, the type of organic materials was reported as the main driver affecting soil fungal community under long-term fertilization. Our research further revealed that the underlying mechanism of affecting the fungal communities in the soils and roots was the differences in phosphorus and zinc contents caused by the application of different organic materials. Therefore, our results highlight that except for phosphorus, zinc content of the organic materials should be considered in long-term organic fertilization systems.

## INTRODUCTION

Fungi are considered the main drivers of plant performance and nutrient cycling (1–3). From bulk soil to the vicinity of plant roots, and even to inside roots, there are rich and diverse fungal communities, corresponding to soil fungi, rhizosphere fungi, and endophytic fungi given their separation by the natural barrier of the rhizosphere systems and plant root tissue (4–6). In bulk soil, the fungi are closely associated with plant phylogeny and ecosystem restoration (7, 8), and they contribute markedly to modulating aspects of soil quality (2), such as organic matter turnover (9), soil aggregate stabilization (10), and element cycling. The fungi dwelling in rhizosphere soil and inside roots (i.e., root-associated fungi) widely participate in plant growth and evolution, linking aboveground biomass dynamics with belowground biota (11, 12). Recruited by host plants, microorganisms generally migrate from the soil into the rhizosphere zone, and ultimately adhere or enter the plant roots (12). Fungi within each habitat are closely related and can cooperate to assist plants in defending against biotic stresses (e.g., pathogens, herbivores), by secreting several volatile organic compounds (13), as well as tolerating abiotic stresses (e.g., heavy metals or drought stress), by strengthening plants’ capacity to uptake soil nutrients (14).

As inherent component of multi-organisms occurring belowground, fungal communities are intrinsically tied to fertilization practices. Many studies have investigated the responses of fungi in bulk soil to fertilization regimes. For instance, mineral fertilization generally reduces soil fungal diversity, enriches some plant fungal pathogens, and disrupts soil structure and functioning (15–18). The application of organic materials, e.g., livestock manures, could improve the ecological interactions, biomass, and activity of soil-dwelling fungi (19, 20). Our earlier study showed that the type of organic matters used can strikingly influence the fungal community composition of bulk soil and decrease the relative abundance of potential pathogens in a 30-year fertilization experiment (9). Besides, some studies have gradually found that rhizosphere fungi can also respond significantly to fertilization measures. Recently Q. Wang et al. (4) found that fungal community of rhizosphere soil was less affected by fertilization (36 years) than that of bulk soil due to rhizosphere effects. Further, organic fertilizer amendments could enrich several fungi like *Mortierella* and *Chaetomium* in rhizosphere soil, which are reportedly nematophagous fungi (21). To date, most research on fertilization impacts on root fungi have mainly focused on specific fungal groups, like arbuscular mycorrhizal fungi (AMF) (22, 23), leaving us with comparatively little knowledge of how long-term fertilization affects overall fungi occupying the root endosphere. Fungi surviving in belowground habitats could be viewed as part of the plant life cycle, whereby fungi residing inside the roots of crops and within the rhizosphere function more to promote plant growth, and vice versa. Therefore, unlike studying a single soil habitat, elucidating the responses of fungal communities in different belowground habitats to long-term fertilization, especially those in root-related habitats (i.e., root endosphere and rhizosphere soil), provides a more powerful way to strengthen our understanding of interactions between fertilization, crops, and belowground fungi.

Under fertilization practices, the factors driving the assembly of and shift in fungal communities living in belowground habitats have been discerned, to some extent. Upon successfully colonizing their habitats, the population growth, survival, and evolution of fungi could be strongly impacted by nutritional status of that habitat (12, 24, 25). Some research has found that fertilization regimes can distinctly modify the rhizosphere and bulk soil physiochemical properties, such as phosphorus, pH, and ions, which then affects the assembly and dynamic of fungal communities within the corresponding habitat (9, 19, 21, 26). On a large scale, the available phosphorus (AP) in soil was the crucial element shaping fungal communities of Andosol agricultural soils under long-term fertilization in Japan (27). Moreover, through elemental fertilization experiments, some studies revealed differential sensitivity of soil fungi to different elements. For example, D. He et al. (17) reported that adding phosphorus, but not nitrogen (N), significantly decreased the fungal richness and altered the fungal community composition of bulk soil on the Qinghai-Tibetan Plateau. More recently, Q. Wang et al. (28) likewise determined that N fertilization (37 years) did not significantly influence the AMF diversity of the rhizosphere, but N combined with P fertilization significantly reduced it. These studies imply a convergent scenario: the responses of fungi to fertilization in belowground habitats could be mainly related to levels of phosphorus. Besides this element, with respect to the ability of fungi to transport nutrients, zinc (Zn) and its absorption is often a research hot spot (29, 30). Zn-deficiency in crop plants is a critical and widespread phenomenon: near 30% of the world’s population is lacking sufficient Zn dietary intake (30, 31). Therefore, if fertilization is capable of triggering a changed Zn content, it seems the impact of this alteration on fungal communities and crops should not be ignored. Yet few studies have sought to clarify the relationships between Zn and root-associated fungi in the context of long-term field applications of organic and mineral fertilizers in agriculture.

The co-occurrence network is a robust tool for exploring interactions within the microbial community (32), whose complexity can be used to comprehensively assess the impact of fertilization upon fungal communities in different belowground habitats. In recent research, we found that long-term fertilization could strongly influence the network complexity of rhizosphere and root AMF by altering the content of phosphorus and carbon (C) in their habitats (23). Through detection of the prominent drivers of microbial community responses to farming practices, we could gain a better understanding and guide the innovative application of traditional farming to staple crops. Yet we have a limited grasp of the factors influencing the diversity, composition and co-occurrence network of total fungi in plant roots under long-term fertilization, and whether the predominant factors involved are consistent with those of non-root habitats (i.e., bulk soil).

Herein, in a wheat field setting, we report on findings from a long-running fertilization (more than 35 years) experiment that includes NPK mineral fertilizer, NPK blended with wheat straw, NPK blended with livestock manures (i.e., pig and cow manure), and no fertilization (control group) to answer these two main questions: (i) How do belowground fungal communities in different habitats, including root-associated habitats (root endosphere and rhizosphere soil) and bulk soil, respond to different fertilization treatments? (ii) Which factors drive these responses in their respective habitats?

## RESULTS

### Variation in the fungal communities of the root endosphere, rhizosphere soil, and bulk soil.

**FIG 1:**
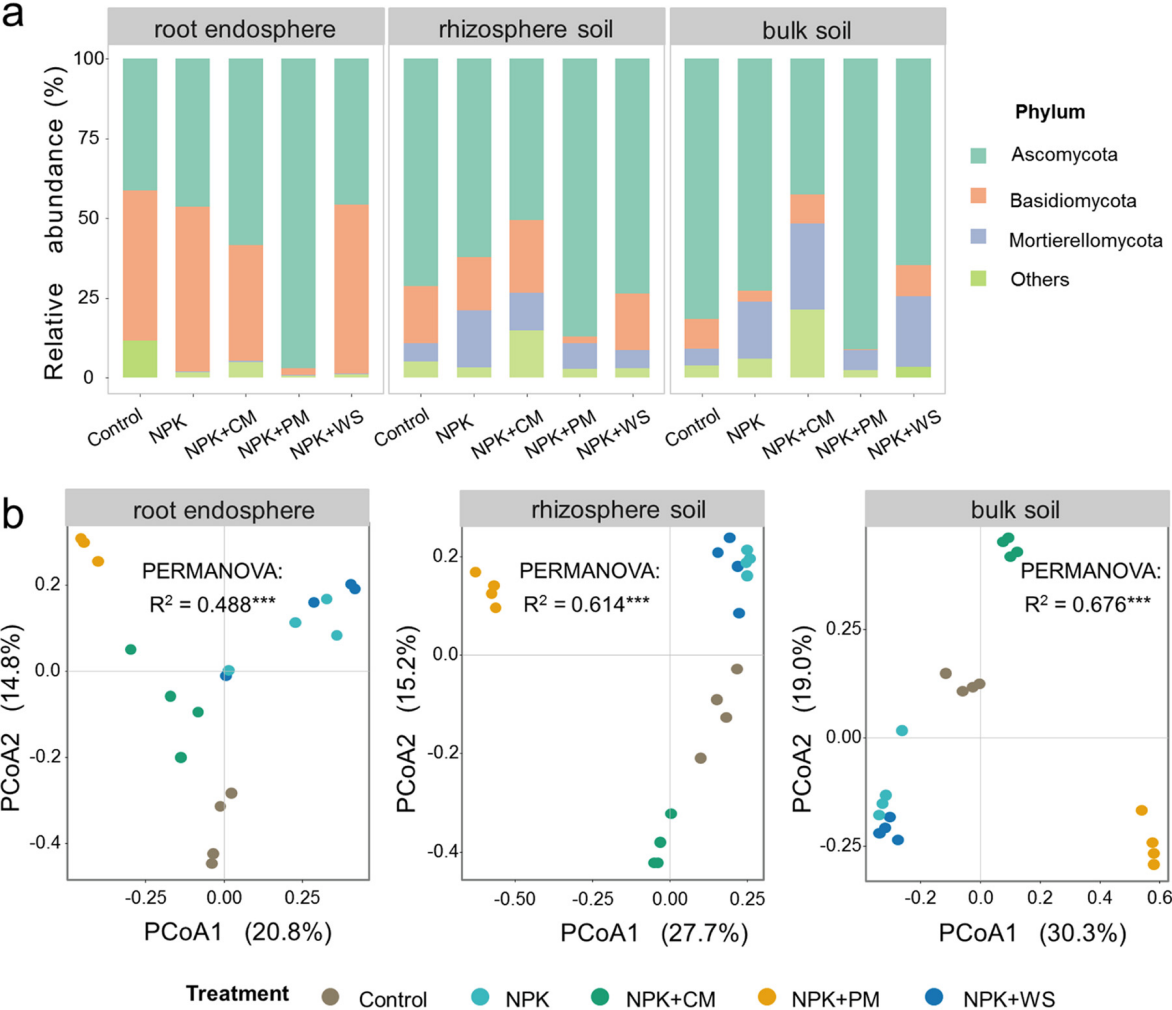
(a) Relative abundance of the dominant fungal phyla and (b) principal coordinates analysis (PCoA) of the variation of fungal communities in root endosphere, rhizosphere soil, and bulk soil. Permutational analysis of variance (PERMANOVA) shows the effects of fertilization treatments on fungal communities.

**FIG 2:**
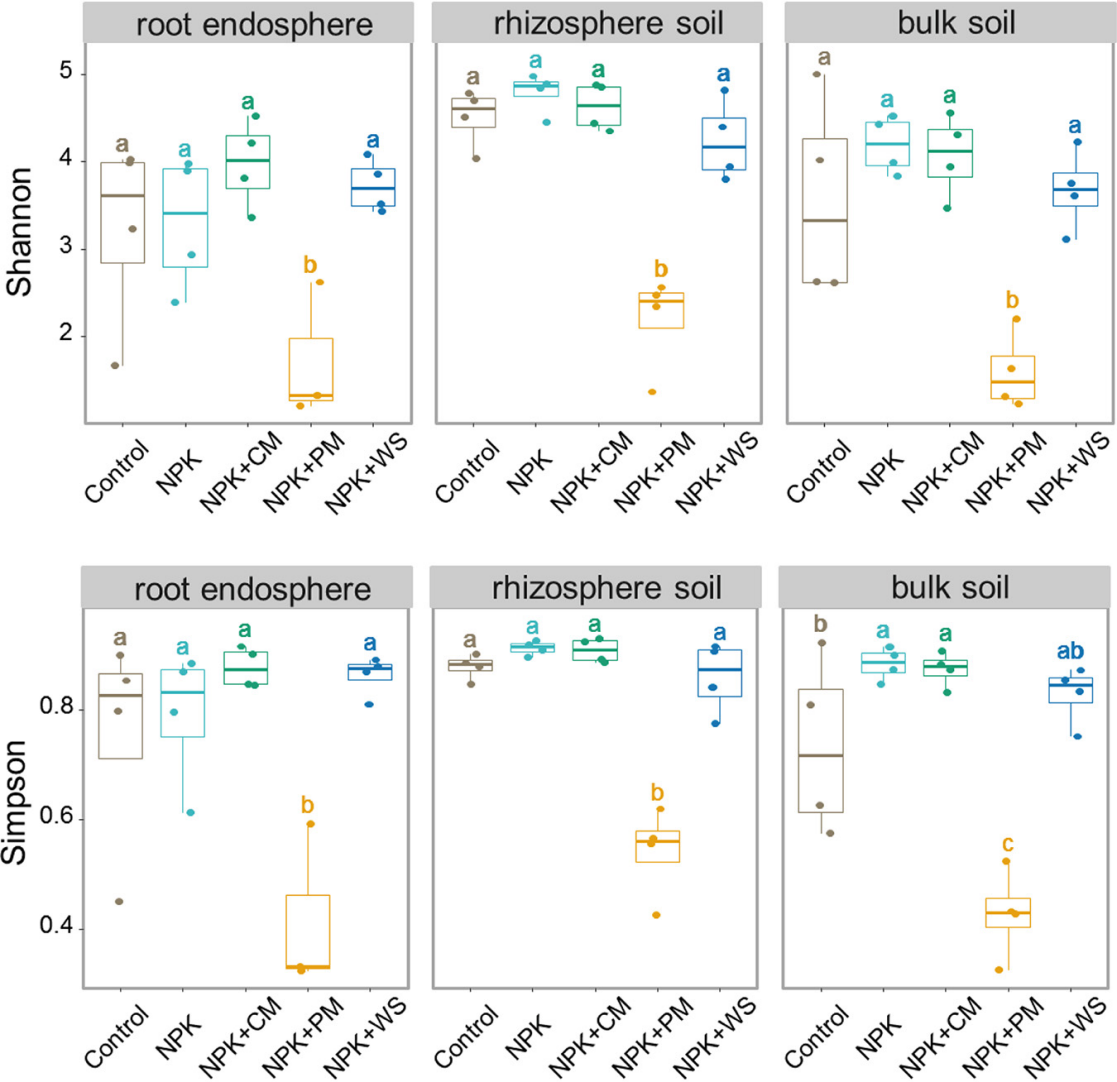
Comparison on alpha diversity of fungal communities among different fertilization treatments in root endosphere, rhizosphere soil, and bulk soil. Values in the columns that do not share the same letter differ significantly (Duncan’s test, *P < *0.05).

Long-term fertilization treatments contributed more to fungal community differentiation than did the belowground habitats (Fig. S2, Table S3). With greater horizontal distance from wheat roots, the fertilization impact on fungal community composition successively increased (root endosphere: R^2^ = 0.488; rhizosphere soil: R^2^ = 0.614; bulk soil: R^2^ = 0.676) (Fig. 1). Pairwise Adonis results revealed that fungal communities were mostly different among treatments within each habitat, in that the NPK+PM treatment generally differed most from either NPK or NPK+WS (Table S4) and also had the significantly lowest alpha diversity among the five treatments in every habitat (Fig. 2).

At the phylum level, Ascomycota, Basidiomycota, and Mortierellomycota dominated the fungal community, irrespective of the treatments and habitats, together accounting for over 80% of the total sequences obtained. Compared with the other treatments, NPK+PM treatment had the highest relative abundance of Ascomycota (96.9%, 87.1%, and 91.1% in root endosphere, rhizosphere soil, and bulk soil, respectively; Fig. 1a, Table S5). Under no-PM treatments (i.e., NPK, NPK+WS, and NPK+CM), the relative abundance of Mortierellomycota in rhizosphere soil and bulk soil apparently increased, while this phylum was scarce in the root endosphere (Fig. 1a, Table S5). At the class level, NPK + PM and NPK treatments significantly increased the relative abundances of Pezizomycetes and Eurotiomycetes, respectively, in rhizosphere soil and bulk soil, while these two classes were almost undetectable in root endosphere (Table S6). Intriguingly, NPK+PM treatment evidently decreased the relative abundance of Sordariomycetes in rhizosphere soil and bulk soil, yet significantly increased it in root endosphere (Table S6). At the ASV level, we found that the relative abundances of eight, 16, and 15 of the 50 abundant fungal ASVs differed significantly among the various treatments in root endosphere, rhizosphere soil, and bulk soil, respectively (Fig. S4). For example, NPK+PM treatment significantly enriched ASV1069 (*Microdochium*) vis-à-vis the other fertilization treatments and NPK+CM significantly promoted the relative abundances of ASV1074 (*Hyalodendriella betulae*), ASV1322 (*Basidiobolus*), and ASV340 (*Subulicystidium*) in root endosphere when compared with other treatments (Fig. S4a). In rhizosphere soil, the relative abundances of ASV1300 (*Tricharina*) and ASV759 (*Eleutherascus*) were significantly higher under NPK+PM than other treatments, while ASV1590 (*Linnemannia*) reached the highest relative abundance under NPK+CM. The relative abundances of ASV1591 (*Chaetothyrium*) and ASV600 (*Knufia*) were significantly increased by NPK, and ASV223 (*Chaetomium*) was significantly enriched by NPK+WS in rhizosphere soil (Fig. S4b). In bulk soil, NPK+PM significantly increased the relative abundances of ASV1300 (*Tricharina*) and ASV759 (*Eleutherascus*), whereas NPK+CM significantly improved those of ASV1590 (*Linnemannia*) and ASV1004 (*Linnemannia*), while under NPK the relative abundances of ASV1256 (*Alternaria alstroemeriae*), ASV727 (*Nothodactylaria*), ASV949 (*Chaetothyrium*), and ASV99 (Fusarium *guttiforme*) were all significantly greater than under the manure-addition treatments (Fig. S4c). In terms of potential trophic types, that of Pathotroph-Saprotroph was apparently enriched by NPK treatment (4.7%∼18.2%) when compared with Control in root endosphere or bulk soil. NPK+PM treatment (0%∼1.4%) sustained much lower relative abundance of Pathotroph-Saprotroph than did either NPK (4.7%∼18.2%) or NPK+WS (4.2%∼10.2%) in all three habitats, especially in root endosphere, and it had the lowest Saprotroph abundance (1.9% at most) among all treatments (Fig. S3).

### Drivers of fungal communities in root endosphere, rhizosphere soil, and bulk soil.

Aiming to distinguish the most powerful predictors for shaping fungal communities across the different fertilization treatments, several physiochemical properties were measured. Compared with Control and NPK treatment, NPK+PM and NPK+CM treatments fostered higher AP, total phosphorus (TP), Zn, and pH in rhizosphere soil and bulk soil as well as higher TP and Zn in root endosphere (Table S2). We first explored the correlation between alpha diversity and physiochemical variables. The random forest analysis revealed that phosphorus and Zn always were the best predictors of diversity changes in the three belowground habitats (Fig. S5). There were significant correlations between phosphorus and alpha diversity in root endosphere, and between Zn and alpha diversity in rhizosphere soil and bulk soil; the sole exception was that between TP and Shannon index in the root endosphere (Table 1). Further, in root endosphere, TP had significant and maximal relationship with fungal community dissimilarity (Mantel test: 0.418), with Zn also significantly related to this fungal community dissimilarity. In rhizosphere soil and bulk soil, AP, TP, and Zn consistently explained more variation in fungal community composition than any of the other factors (Mantel test: > 0.45) (Table 2). These results were corroborated by the MRM (Table S7) and random forest analysis (Fig. S6).

**TABLE 1:** Correlations between physiochemical variables and fungal diversity in three habitats^*a*^

Root endosphere			Rhizosphere soil			Bulk soil		
Variable	Shannon	Simpson	Variable	Shannon	Simpson	Variable	Shannon	Simpson
Zn	–0.634**	–0.725***	Zn	–0.872***	–0.883***	Zn	–0.730***	–0.772***
Na	–0.550*	–0.621**	AP	–0.801***	–0.832***	AP	–0.671***	–0.672***
TP	–0.368	–0.477*	TP	–0.702***	–0.704***	TP	–0.540*	–0.525*
Mg	–0.269	–0.319	NO_3_^−^-N	–0.400	–0.430	Ca	–0.330	–0.372
Fe	–0.215	–0.235	C:N	–0.219	–0.202	C:N	–0.240	–0.206
TN	–0.192	–0.295	DON	–0.193	–0.160	pH	–0.159	–0.246
TK	–0.142	–0.184	NH_4_^+^-N	–0.152	–0.178	DOC	–0.087	–0.013
Mn	–0.096	–0.059	DOC	–0.109	–0.090	TC	–0.001	0.052
Ca	–0.078	–0.212	Moisture	–0.093	–0.078	DON	–0.001	0.064
TC	0.012	0.001	TN	–0.087	–0.049	TN	0.015	0.076
C:N	0.195	0.302	TK	–0.078	–0.072	Mg	0.114	0.006
			AK	–0.033	0.005	Moisture	0.118	0.163
			pH	–0.006	–0.008	Na	0.138	0.065
			TC	0.016	0.046	NH_4_^+^-N	0.143	0.188
			Mn	0.073	0.081	Mn	0.162	0.097
			Mg	0.128	0.160	NO_3_^−^-N	0.187	0.261
			Ca	0.130	0.120	AK	0.238	0.279
			Na	0.276	0.274	TK	0.332	0.392
			Fe	0.326	0.363	Fe	0.364	0.261

a AP, available phosphorus; AK, available potassium; TC, total carbon; TN, total nitrogen; TP, total phosphorus; TK, total potassium; C:N, total carbon/total nitrogen; DOC, dissolved organic carbon; NO_3_^−^-N, nitrate; NH_4_^+^-N, ammonium; DON, dissolved organic nitrogen; Ca, calcium; Mg, magnesium; Na, sodium; Fe, iron; Mn, manganese; Zn, zinc. Each physiochemical property is measured from its respective habitat. *, *P < *0.05; **, *P < *0.01; ***, *P < *0.001.

**TABLE 2:** Results of the Mantel test analysis between physiochemical variables and community composition of fungi in three habitats^*a*^

Root endosphere		Rhizosphere soil		Bulk soil	
Variable	Statistic r	Variable	Statistic r	Variable	Statistic r
TP	0.418***	TP	0.648***	AP	0.643***
Zn	0.192*	AP	0.559***	TP	0.632***
TC	0.085	Zn	0.463***	Zn	0.473***
TN	–0.007	pH	0.387***	pH	0.438***
TK	0.243**	AK	0.371***	AK	0.317***
C:N	–0.022	TC	0.371***	TC	0.406***
Ca	0.201*	TN	0.431***	TN	0.462***
Mg	0.081	TK	0.026	TK	0.257***
Na	0.019	C:N	0.117	C:N	0.134
Fe	0.055	Ca	–0.016	Ca	0.158*
Mn	0.129	Mg	0.085	Mg	0.075
		Na	0.263**	Na	–0.011
		Fe	0.115	Fe	0.093
		Mn	–0.045	Mn	–0.079
		Moisture	0.169*	Moisture	0.213*
		DOC	0.24**	DOC	0.211*
		DON	0.05	DON	0.006
		NO_3_^−^-N	0.229**	NO_3_^−^-N	0.034
		NH_4_^+^-N	–0.044	NH_4_^+^-N	–0.09

a TP, total phosphors; AP, available phosphorous; Zn, zinc; AK, available potassium; TC, total carbon; TN, total nitrogen; TP, total phosphorus; TK, total potassium; C:N, total carbon/total nitrogen; DOC, dissolved organic carbon; NO_3_^−^-N, nitrate; NH_4_^+^-N, ammonium; DON, dissolved organic nitrogen; Ca, calcium; Mg, magnesium; Na, sodium; Fe, iron; Mn, manganese. Each physiochemical property is measured from its respective habitat. nperm = 999; *, *P < *0.05; **, *P < *0.01; ***, *P < *0.001.

Given that phosphorus and Zn exhibited incomparable roles in shaping fungi attributed to long-term fertilization at the community level, this inspired us to probe the effects of each element on fungi at conventional and finer taxonomic levels, namely, phylum, class, ASV, and potential trophic types. First, considering Ascomycota, it had a positive and highest correlation with TP (*rho *= 0.518) in root endosphere only. Basidiomycota was negative correlated with TP in root endosphere (*rho* = −0.461) and with phosphorus in bulk soil (AP: *rho* = −523; TP: *rho =* –0.473) (Table S8). Second, major fungal classes were found significantly correlated with both phosphorus and Zn contents. For instance, the correlation between the relative abundance of Sordariomycetes and P was strongly positive in root endosphere, but negative in both rhizosphere soil and bulk soil (Table S9). The relative abundance of Pezizomycetes showed a positive relationship with AP only in rhizosphere soil yet with Zn in all three habitats (Table S9). The relative abundance of Eurotiomycetes was negatively correlated with AP and TP in bulk soil, and with Zn in both rhizosphere soil and bulk soil (Table S9). Third, several abundant fungal taxa demonstrated various responses to key factors. In root endosphere, the relative abundances of ASV1069 (*Arthropsis truncata*) and ASV1074 (*Hyalodendriella betulae*) had positive correlation with TP (Table S10). In rhizosphere soil, the AP and Zn correlations with the relative abundance of ASV727 (*Nothodactylaria*) were negative; however, those with ASV1590 (*Linnemannia*), ASV1300 (*Tricharina*), and ASV759 (*Eleutherascus*) were all positive (Table S10). In bulk soil, the relative abundances of *Linnemannia* (ASV1590 and ASV1004) exhibited strong positive relationships with AP and Zn yet negative ones for ASV1256 (*Alternaria alstroemeriae*), ASV727 (*Nothodactylaria*), ASV949 (*Chaetothyrium*), and ASV99 (Fusarium *guttiforme*) (Table S10). The pH was also strongly related with some abundant taxa; for example, negative correlation: ASV99 (Fusarium *guttiforme*), positive correlation: ASV1590 (*Linnemannia*) (Table S10). Lastly, long-term fertilization generally augmented phosphorus and Zn contents, which had strong negative correlations with the relative abundances of potential phytopathogens (Fig. S7, S8).

### Co-occurrence networks of fungal communities in root endosphere, rhizosphere soil, and bulk soil.

**FIG 3:**
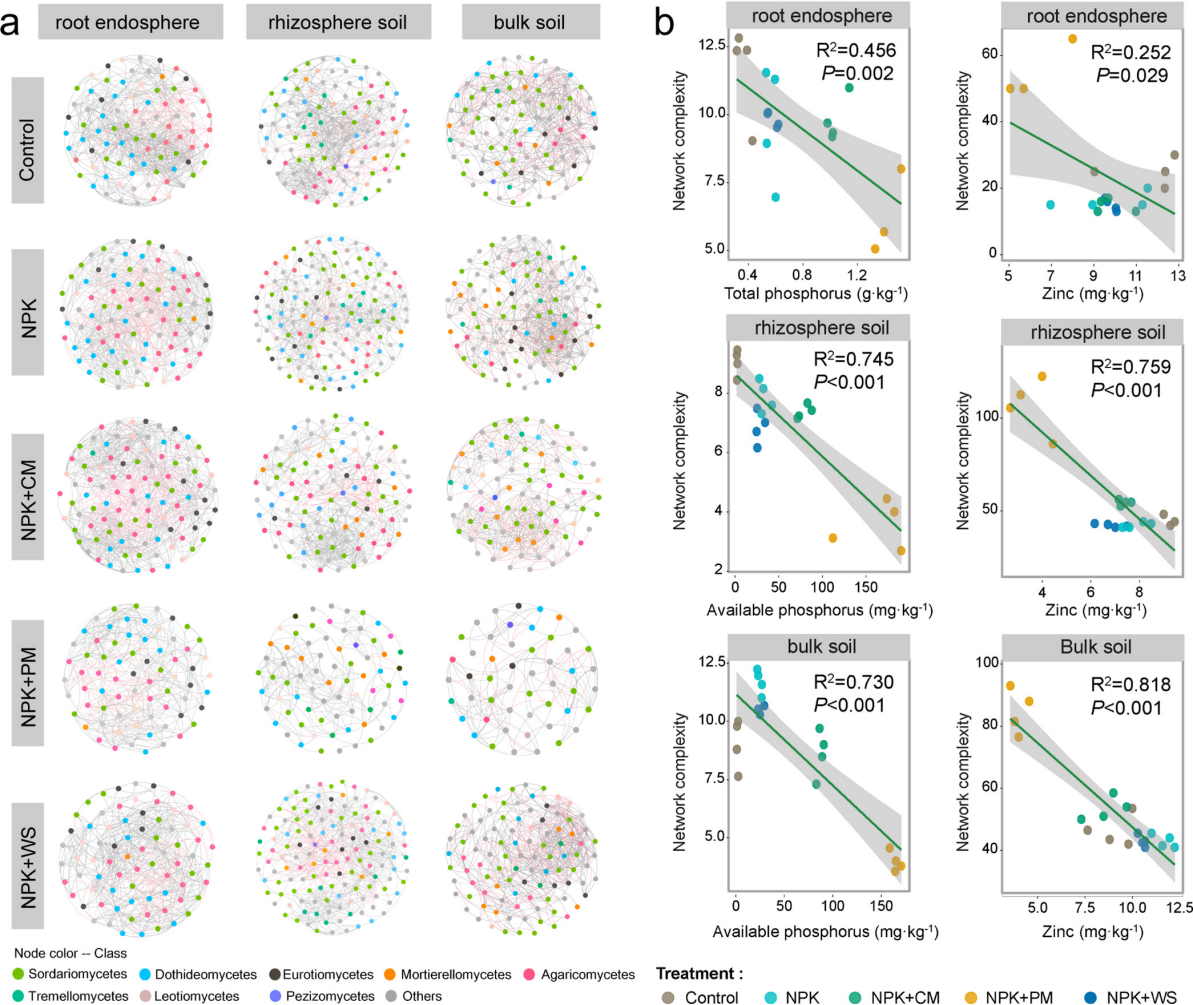
Fungal co-occurrence network patterns among different fertilization treatments and habitats. (a) The sub-network in root endosphere, rhizosphere soil and bulk soil. (b) The relationships between the phosphorus and zinc contents and the network complexity (represented by average degree) in the three habitats. Data are fitted using linear regression. Each physiochemical property is measured from its respective habitat.

Using all samples from root endosphere, rhizosphere soil, and bulk soil, the corresponding overall co-occurrence networks of fungal communities were constructed. These respectively consisted of 136 nodes and 1,435 edges, 234 nodes and 1,666 edges, and 202 nodes and 1,637 edges (Fig. S9). Due to the significant variation in fungal community structure across the five treatments, we analyzed the sub-network for each treatment separately (Fig. 3). Interestingly, the manure mixed treatments, especially NPK+PM having the lowest number of nodes and average degree, simplified the co-occurrence relationship of networks (Fig. 3). To further explore how physiochemical variables might impact fungal network complexity, we examined correlations between these variables and typical network characteristics (i.e., average degree, edges, and nodes). Random forest analysis revealed that phosphorus and Zn were almost always important predictors of alterations in these characteristics in the soil habitats, with TP being the best predictor of shifts in edges and average degree in the root endosphere (Fig. S10). Accordingly, we fitted regressions between key factors and these characteristic values, finding significant and negative linear relationships for average degree versus TP content (R^2^ = 0.456, *P = *0.002) and Zn content (R^2^ = 0.252, *P = *0.029) in root endosphere (Fig. 3b), likewise, between average degree and both AP content in soil habitats (rhizosphere soil: R^2^ = 0.745, *P < *0.001; bulk soil: R^2^ = 730, *P < *0.001) and Zn content (rhizosphere soil: R^2^ = 0.759, *P < *0.001; bulk soil: R^2^ = 0.818, *P < *0.001). Other factors, like pH, also significantly influenced these characteristics to some extent (Table S11). Overall, increasing phosphorus and Zn contents arising from long-term fertilization might be leading factors contributing to the reduced network complexity of fungal communities in root endosphere, rhizosphere soil, and bulk soil of wheat fields.

## DISCUSSION

### Fungal communities are affected less by habitat, and more by long-term fertilization.

It has been widely found that both agricultural fertilization and habitats can impact belowground microbial communities (9, 33, 34). In our study, the effect of long-term fertilization on the fungal community surpassed that of habitats, but gradually diminished when going from bulk soil to root endosphere (Fig. 1). This could be explained by the fact that despite long-term fertilization having exerted a more powerful impact on fungal communities, as the fungi get closer to the roots of the crop, the selection of the crop itself become stronger, which buffers the intense influence of fertilization (4, 5). For instance, most members of the Pezizomycetes are soil saprophytes (35, 36), and Mortierellomycetes are capable of solubilizing phosphorus from soil and thereby increase the AP in soil solution (27). In our study, both taxa were mainly enriched in rhizosphere soil and bulk soil under fertilization, but not so in root endosphere (Table S6). Actually, root endosphere harbored more Agaricomycetes, a result consistent with studies that reported numerous Agaricomycetes, which can function ecologically as saprophytes as well as a mutualists, being detected as plant endophytes (37–39). Besides, there were fertilizer-oriented fungi prevalent in each habitat. For taxonomy species, some species have different preferences for fertilizers. The NPK+PM treatment enriched more Pezizomycetes than other treatments in the three habitats, while the NPK treatment obviously increased the relative abundance of Eurotiomycetes than other treatments in soils (Table S6). For potential functional guilds, R. Sun et al. (9) found that treating the soil with NPK could augment the growth of potential pathogens, while NPK+PM treatment inhibited their enrichment to a certain extent in bulk soil. That differential effect was confirmed by our results, but we also found that it was applicable to fungi in rhizosphere soil and root endosphere (Fig. S3). This shows that although the rhizosphere and root system barriers may buffer stimulation of their fungal communities from long-term fertilization, this common agricultural practice can nonetheless significantly impact the fungi occupying the crop’s rhizosphere and even its roots—an effect having certain similarities. In short, fungal communities in different belowground habitats seem to respond similarly to fertilization.

### Phosphorus and zinc contents are consistent drivers of fungal diversity in soil and root habitats.

We have found that the assembly and alteration of fungal community is strongly induced by the physiochemical properties of its immediate environment (12, 26). Here, we found changed phosphorus and Zn contents were the paramount factors driving the reduction in fungal diversity in each habitat under long-term different fertilization (Table 1, Fig. S5). This finding is partly in line with other research reporting that manure applications reduced the diversity of soil fungal community when compared with mineral fertilization, along with a significant negative correlation with phosphorus content (17, 27). Generally, there exists a considerable concentration gradient of phosphorus spanning the rhizosphere from bulk soil to the root surface, a consequence of this element’s poor solubility and mobility in soils (40, 41). Plants thus appeal to microorganisms, especially fungi, to assist them with the acquisition of phosphorus by extending the absorption surface and mediating this nutrient’s dissolution (40). In our study, manure applications modified the belowground habitats’ nutrient levels (e.g., more phosphorus resources, more Zn) more effectively than either mineral fertilization (NPK) or the wheat straw (WS) application treatment (Table S2). It may be due to the fact that the manure itself brings in abundant phosphorus (Table S1), and almost 70% of TP in manure is unstable and contributes to high phosphorus availability and mobility within soil solutions (41, 42). Remarkably, pig manure had higher phosphorus content than cow pig manure and after adding pig manure, the phosphorus content of soils and wheat roots was significantly higher than that of cow manure addition (Table S1, Table S2). Hence, long-term fertilization, especially pig manure application, can supply adequate and readily accessible phosphorus and phosphorus-like nutrients across soil to roots, which may lessen the dependence of crops on microbiota, thereby lessening total fungal diversity probably. Besides, phosphorus-rich habitats tend to induce more competition among belowground organisms. Such places could be more hospitable to bacteria, whose higher diversity and abundance here (43, 44) leaves co-occurring fungi competing with them to survive. Also, the abundance of soil fungivores generally increases under organic manure applications (45, 46), which is probably correlated with phosphorus content (17, 47), and their greater presence could exercise selective pressures on soil fungal community leading to reduced fungal diversity over time.

Moreover, phosphorus is pivotal for the signal transduction pathway regulating the intake and translocation of Zn within the soil to plant, and vice versa (48, 49). Often, the Zn concentration in soil solution is too low to meet what is demanded by plants, so the primary way of supplying Zn to them may be its dissemination within the rhizosphere habitat (30, 50). Although we did not measure the zinc content of the manure additives, Nicholson et al. (51) have estimated that over the total agricultural area of England and Wales, livestock manures are responsible for around 40% of the total inputs of Zn, and our results showed that NPK+PM obviously and significantly increased the Zn content in bulk soil and rhizosphere soil than other fertilization treatments (NPK+CM, NPK+WS, and NPK) (Table S2). It means that the addition of pig manure can greatly increase the Zn content in the farmland soil, thereby meeting the needs of crop growth. Based on this, similar to phosphorus, it is not unreasonable to link the competition for resources and survival among fungal communities to Zn-induced changes in fungal diversity. Along with these aspects, high Zn content itself might limit the reproduction of certain fungi such as Sordariomycetes and Leotiomycetes (Table S9). This could happen via extreme sensitivity to heavy metals, including Zn, during fungal reproduction rather than mycelial growth, which could decrease the production of conidia considerably (52, 53). Meanwhile, Zn is among the recognized key players in host–pathogen interactions; for instance, wheat plants with higher Zn content incurred less *Rhizoctonia* root rot (30, 54, 55). Our results also show that Zn may suppress the growth of potential phytopathogens in belowground habitats (Fig. S8). All these described processes could enable a high Zn content brought by pig manure addition to cause fungal diversity declines in belowground habitats. Taken together, as phosphorous and Zn contents rise in response to long-term fertilization and the diversity of soil fungi falls, this contrasting dynamic may drive the differential assembly and alteration of microbes in rhizosphere and bulk soil, thereby modulating their recruitment into crop roots (56).

### Phosphorus and zinc contents jointly affect fungal community composition.

In the study, the altered phosphorus and Zn contents induced by long-term different fertilization contributed most to the variation in fungal community composition of each habitat (Table 2, Table S7, Fig. S6). We can try to explain this concretely from two aspects. First, the presence and abundance of some nutrient-related fungi are closely related to levels of phosphorus and Zn in their respective habitats. In rhizosphere soil and bulk soil, phosphorus showed positive correlations with the relative abundances of several taxa belonging to Mortierellomycetes (e.g., ASV1004, ASV1590), which have phosphate-solubilizing activity that increase the amount of available phosphorus in soil (27). These taxa also had positive relationships with Zn, implying their importance in the dissolution and migration of Zn within soil solution. In any case, saprophytic fungi can decompose organic matter and increase the amount of nutrients in the soil available for uptake by plants (57). Thus, it is not surprising that the saprophytes’ growth was restricted when their occupied zones had enough or even excess available nutrients brought on by long-term fertilization (Fig. S3, Fig. S7). Specifically, *Deconica* (ASV813) in root endosphere (58) and *Myrmecridium* (ASV1467) in rhizosphere soil (59), both usually regarded as saprophytes, were negatively associated with phosphorus. Interestingly, Zn had a consistently positive correlation with the relative abundance of Pezizomycetes in all three habitats, a class significantly enriched by NPK+PM treatment (Table S6). Most Pezizomycetes are widely known as a kind of saprophytes (35, 36), and we may speculate that several taxa of this class (i.e., *Tricharina* [ASV1300] and *Eleutherascus* [ASV759]) might support the Zn absorption of plants in belowground habitats having high phosphorus levels, to relieve and avoid the harm of Zn-deficiency in crops.

Second, after long-term manure applications, the possible process of inhibiting the potential pathogens and enriching the anti-pathogens may contribute to the healthy growth of crops (9, 60). In our study, phosphorus and Zn showed strong negative correlations with some potential phytopathogens in their respective habitats under long-term fertilization (Fig. S7, S8), such as *Alternaria alstroemeriae* (ASV1256) in bulk soil reported as a notorious generalist pathogen capable of infecting more than 100 host plants (61, 62). Fusarium *guttiforme* (ASV99) in bulk soil is a facultative or obligate plant pathogen and also acts as an opportunist (63, 64); *Marasmius* (ASV309) in rhizosphere soil is a saprophytic and pathogenic fungus (65); *Microdochium* (ASV1067) in root endosphere was recently shown to cause pink snow mold, grass decay, leaf spots, and head blight (66). Lastly, positively correlated with TP in the root endosphere (Table S10) was *Arthropsis truncate* (ASV1069), whose metabolites could be responsible for antagonistic interactions with specific pathogens (67). Thus, the increase in phosphorus and Zn contents caused by long-term mixed fertilizer application with manures may steer shifts in fungal community composition in each habitat toward a state that is more beneficial to crop growth.

### Phosphorus and zinc contents are closely tied to fungal network complexity.

Long-term, stable fertilization practices may gradually simplify the microbial community in an almost unchanged planting system, but it seems to be more beneficial to crop growth (68, 69). This domestication could depend on the metabolism of critical nutrients to the plant growth. As Mendes et al. (70) found, microbes, living in rhizosphere soil with less complicated taxa and functions, are predominant in the metabolic pathways related to phosphorus uptake and alkylphosphonate utilization and other nutrients. In the study, by constructing the co-occurrence networks of fungal communities, we found that the lower network complexity of the fungal community in each habitat was correlated chiefly with the increased phosphorus and Zn contents under long-term fertilization management (Fig. 3, Fig. S10). Therefore, we speculate that the NPK+PM treatment might trigger more specialized specific metabolic pathways to become activated in belowground habitats, especially those involving phosphorus and Zn, than the NPK or NPK+WS treatments, despite the lower complexity of its fungal co-occurrence network. Considering the lower fungal diversity in the treatments of mineral fertilizer mixed with manures, their co-occurrence networks’ lower complexity is certainly not surprising. The finding complements a recent study that demonstrated long-term fertilization can affect the diversity and network complexity of AMF communities in the rhizosphere soil and root endosphere by affecting phosphorus and pH levels (71). However, the earlier work by Ling et al. (72) showed that the long-term addition of organic fertilizers generates a more complex co-occurrence network of bacteria when compared with adding mineral fertilizers alone. Therefore, the benefits of a combined application of organic fertilizers to microbial diversity and ecological interactions should attach a specific-group restriction, called context-dependent.

To sum up, fungal community composition in bulk soil under long-term mineral fertilization is substantially affected by the type of organic matter applied (9). Building on that, this study found that root-associated fungal communities (in root endosphere and rhizosphere soil) are also significantly influenced by long-term fertilization in terms of their diversity, composition and the complexity of their co-occurrence networks. Importantly, these effects were mainly due to changes in phosphorus and Zn contents induced by different types of organic matter inputted, in which the participation of several fungal species may prevail. However, going from soils to rhizosphere to the interior of plants, the absorption and release of phosphorus or Zn involves various complex components and physiochemical reactions, generating intricate forms of either element. What roles specific fungi or microorganisms play in these transformation processes are intriguing and rewarding avenues for further fruitful investigation. It is noteworthy that the above potential interactivity between these two elements and fungal microbes is not independent of soil pH, in that soil pH not only has important direct and indirect impacts upon fungal community dynamics (9, 56, 73, 74), but is also closely tied to the speciation and concentration of Zn and phosphorus forms within soils. Nonetheless, long-term fertilization significantly affects fungal communities may be the result of compounding effects of multiple environmental factors or undetected factors in fertilizers and habitats that are relevant to microbes, and it is worthy doing further experiments to test.

### Conclusion.

Long-term fertilization (35 years) strongly affects the diversity, community composition, and co-occurrence pattern of fungal communities in root endosphere, rhizosphere soil, and bulk soil habitats in a wheat field. In particular, the combined application of NPK fertilizers and pig manure greatly decreases both fungal diversity and the complexity of co-occurrence networks. Crucially, the diversity, community composition, and network complexity of belowground fungal communities are strongly associated with phosphorus and Zn contents in all three habitats. Our results indicate that long-term fertilization not only can impact fungal communities in soils but also in root endosphere, and this is strongly associated with the contents of phosphorus and Zn. These findings suggest a possible way to regulate fungal communities and their functioning predictably, by adjusting the levels of phosphorus and Zn in soils and roots of crops through tailored agricultural fertilization management practices.

## MATERIALS AND METHODS

### Experimental design, sample collection, and physiochemical analysis.

The ongoing experiment—subjected to a wheat-soybean rotation since 1982—is located in Mengcheng, Anhui Province, China (33°13′ N, 116°35′ E), whose climate is seasonal temperate semi-humid monsoon. Annual precipitation is 872 mm and annual mean temperature is 14.8°C. The soil type is the lime concretion black soil. Five treatments, each with four replicates (plots), were arranged in a completely randomized block design (each plot is 70 m^2^): (i) Control: no fertilization; (ii) NPK: chemical fertilizers comprising urea (180 kg N ha^−1 year−1^), superphosphate (90 kg P_2_O_5_ ha^−1 year−1^), and potassium chloride (86 kg K_2_O ha^−1 year−1^); (iii) NPK+CM: NPK mineral fertilizers plus cow manure (about 30,000 kg/hm^2^); (iv) NPK+PM: NPK mineral fertilizers plus pig manure (about 15,000 kg/hm^2^); (v) NPK+WS: NPK mineral fertilizers plus wheat straw (about 7500 kg/hm^2^). Each fertilizer type was added annually to the soil prior to sowing wheat (Triticum aestivum L.), and mixed well. Wheat root and soil samples were collected at the wheat booting stage (April 20, 2017). The element contents of pig manure, cow manure, and wheat straw are shown in Table S1.

Bulk soil was taken at 12 sampling points from the cultivated layer (0 to 15 cm depth), and 30 healthy wheat plants were randomly selected. The soil adhering to their root surfaces were brushed down, and designated the rhizosphere soil, with the root tissues also collected from each plot. Overall, the collected samples per plot consisted of bulk soil, rhizosphere soil, and roots respectively pooled into sealed polyethylene bags. The composite samples of bulk soil and rhizosphere soil were then passed through a 2-mm sieve to remove any impurities, and all samples were divided into two parts: one stored at 4°C for the physicochemical properties analysis, and the other stored at −40°C for subsequent DNA extractions. Following the methods described in Yang et al. (7), we measured 19 physiochemical properties of bulk soil and rhizosphere soil: pH, moisture, available phosphorus (AP), available potassium (AK), total carbon (TC), total nitrogen (TN), total phosphorus (TP), total potassium (TK), total carbon: total nitrogen ratio (C:N), dissolved organic carbon (DOC), nitrate (NO_3_^−^-N), ammonium (NH_4_^+^-N), dissolved organic nitrogen (DON), calcium (Ca), magnesium (Mg), sodium (Na), iron (Fe), manganese (Mn), and zinc (Zn). For the root samples, 11 physiochemical properties, namely, the TC, TN, TP, TK, C:N, Ca, Mg, Na, Fe, Mn, and Zn of root tissue, were quantified using the methods described by Ma et al. (71). All physiochemical properties are summarized in Table S2.

### Root surface sterilization.

According to the modified ethanol-sodium hypochlorite method detailed by Sun et al. (75), we used several procedures to rid the samples of surface microorganisms: those roots without any obvious soil attachments were successively dipped in 70% ethanol (for 2 min), 2% NaOCl (for 5 min), and 70% ethanol (for 30 seconds), followed by five washes under sterile distilled water and then dried with sterile dry filter papers. To examine the efficacy of this surface sterilization, the final washed water of each sample were spread on potato dextrose agar (PDA) plates and cultured an incubator at a constant temperature and humidity for 7 days. Then, each sample that had been successfully surface-sterilized was ground with liquid nitrogen, using sterile mortars in sterile room, with all of these samples stored in a sterile tube at −40°C until their further processing.

### DNA extraction and amplicon sequencing.

Following manufacturer’s instructions, root DNA was extracted with the DNeasy Plant minikit (Qiagen, Germany), and DNA in rhizosphere soil and bulk soil was extracted using the Fast DNA SPIN Kit (MP Biomedicals, Santa Ana, CA). The primer pair *ITS1F* (5′-CTTGGTCATTTAGAGGAAGTAA-3′)/*ITS2R* (5′-GCTGCGTTCTTCATCGATGC-3′) was used to amplify the *ITS1* rRNA gene fragments for fungi (76). The PCR system consisted of 0.4 μL of TransStart Fastpfu DNA polymerase (AP221-02, TransGen, China), 4 μL of 5×FastPfu buffer, 2 μL 2.5 mM dNTPs, 0.8 μL each of 5 μM forward and reverse primers, 0.2 μL of BSA, 1 μL DNA template (10 ng/μL), and topped with ddH_2_O to 20 μL. The PCR thermal cycling program was 95°C for 3 min, followed by 30 cycles (30 seconds at 95°C, 30 seconds at 52°C, 45 seconds at 72°C), ending with an extension at 72°C for 10 min. The ensuing sequence products were run on the Illumina Mi-Seq PE250 platform (Illumina, Inc., San Diego, CA, USA).

### Bioinformatics analyses.

Raw sequencing data were processed by QIIME2 (77). After undergoing quality-filtering (quality threshold = 30) and trimming (minimum length = 200 bp), these sequences were “denoised” via the DADA2 pipeline (78). In this way, a total of 2,874,840 sequences were obtained from 59 samples (i.e., 20 bulk soils, 20 rhizosphere soils, and 19 root endospheres; one root endosphere sample was lost from the NPK+PM treatment). The phylogenetic tree and species annotations were constructed based on representative sequences, with any non-fungal sequences first removed from the data set. The UNITE fungal ITS database was used for taxonomical assignments (79). All 59 samples were rarefied to 21,464 sequences per sample (minimum sequence number) for the following analyses. Several amplicon sequence variants (ASVs) lacking a taxonomic assignment from QIIME2 were instead identified using BLASTn and the internal transcribed spacer region (ITS) from fungi type searched against a reference material database (80). Further, the functional guilds of fungal trophic type were obtained online from FUNGuild (http://www.funguild.org/) and literature (81). For this, only guild assignments whose confidence ranking was “probable” or “highly probable” were retained for analysis.

### Statistical analysis.

Fungal diversity (Simpson and Shannon indexes) were calculated from the rarefied fungal ASV table using the “phyloseq” R package. To explore which variables most influenced the alpha diversity of fungi, random forest analysis—a powerful machine learning tool, generating high prediction accuracy by employing an ensemble of decision trees based on bootstrapped samples from a data set in the “randomforest” and “rfPermute” R packages (82, 83)—was conducted with 999 permutations. For each candidate predictor, an importance value was then derived to winnow the predictors by using the *importance* and *varImpPlot* functions. The factors whose significance was less than 0.01 were selected further as the “optimal predictors,” according to the increase in node purity and mean square error values, in the “randomForestExplainer” package (56, 84). Finally, correlations between these optimal predictors and fungal diversity under the five fertilization treatments were calculated.

To visualize the variation within and among fungal community composition, a principal coordinates analysis (PCoA) was implemented based on the Bray–Curtis dissimilarities. To assess the effects of fertilization and habitat upon fungal community composition, permutational multivariate analysis of variance (PREMANOVA) was performed with 999 permutations in the “vegan” R package. Meanwhile, another PERMANOVA (999 permutations) was run to compare the community differences between any pair of fertilization treatments in each habitat using the “vegan” package. The effect of physiochemical variables on structuring fungal community was explored in depth, by calculating the correlation between the two distance matrices whose significance was determined via permutation testing implemented with *mantel* function in the “vegan” package (85). We also conducted multiple regression analysis on matrices (MRM), which can generally fit and assess linear, nonlinear, or nonparametric relationships between a multivariate response distance matrix and any number of explanatory distance matrices (86, 87) to determine the relative contribution of each physiochemical property. Before the MRM’s execution, variable clustering was performed to identify any redundant environmental variables, using the *varclus* function in the “Hmisc” package. Those variables found to be strongly correlated (Spearman’s *rho *> 0.6) were removed from the MRM procedure (Fig. S1). Fungal community dissimilarity (based on Bray–Curtis distances) was ln-transformed, while all matrices of physiochemical factors (based on Euclidean distances) were standardized using the *stdize* function in the “MuMIn” package (88). We ran the MRM analysis by applying the *MRM* function in the “ecodist” package (89). The results of the Mantel test and MRM analysis were then confirmed by the random forest analysis (described above). Using the *corr.test* function in the “psych” package, correlations were computed between physiochemical variables and the relative abundance of fungal community members (including major phyla, major classes, and several taxa with significant difference of top 50 abundant, ASVs accounted for 71.3% of the total sequence and potential trophic-types).

Co-occurrence patterns in each habitat were constructed using CoNet plug in Cytoscape v3.4.0 and visualized in Gephi (http://gephi.github.io/). To achieve higher reliability and precision, those ASVs with relative abundances < 0.005% were detached, and the relationship among ASVs was examined using Pearson correlation, Spearman correlation, Bray–Curtis dissimilarity, and Kullback–Leibler dissimilarity synchronously accompanied with *P*-values calculated via permutation testing and being adjusted by Benjamini–Hochberg’s correction (90, 91). Sub-networks were then generated from the whole networks by preserving the ASVs presented in each sample, using the *subgraph* function in the “igraph” package. Topological characters (nodes, edges, and average degree) were used to evaluate the complexity of each sub-network (56, 91). Random forest analysis was also conducted to discriminate the most important predictors influencing the complexity of each fungal co-occurrence network. The influence of optimal predictors on networks’ topological characters was quantified with linear regression models in R software.

### Data availability.

All raw sequence data for *ITS* genes were deposited at the National Center for Biotechnology Information (NCBI) under the Sequence Read Archive (SRA) accession number PRJNA801083 (BioSample accession numbers SAMN25294183 to SAMN25294241), which are publicly available at https://www.ncbi.nlm.nih.gov/sra/PRJNA801083.
