# Supplementary material for: Phosphorus and Zinc Are Strongly Associated with Belowground Fungal Communities in Wheat Field under Long-Term Fertilization
Source: Microbiol Spectr. 2022 Mar 10;10(2):e00110-22. doi: 10.1128/spectrum.00110-22 (PMC9045391; doi:10.1128/spectrum.00110-22)
Supplement: SUPPLEMENTAL FILE 1 — Supplemental material. Download SPECTRUM00110-22_Supp_1_seq4.pdf, PDF file, 1.7 MB [file spectrum00110-22_supp_1_seq4.pdf]

Supplementary Materials

Journal name: Microbiology Spectrum

Phosphorus and zinc are strongly associated with belowground fungal communities in wheat field under long-term fertilization

Di Wu [a,b], Yuying Ma [a], Teng Yang [a,b], Guifeng Gao [a], Daozhong Wang [c], Xisheng Guo [c], Haiyan Chu [a,b]#

[a]State Key Laboratory of Soil and Sustainable Agriculture, Institute of Soil Science, Chinese Academy of Sciences, 71 East Beijing Road, Nanjing 210008, China

[b]University of Chinese Academy of Sciences, Beijing 100049, China

[c]Key Laboratory of Nutrient Cycling and Resources Environment of Anhui Province, Soil and Fertilizer Research Institute, Anhui Academy of Agricultural Sciences, 40 South Nongke Road, Hefei 230001, China

Running Head: Effects of long-term fertilization on fungal community

#Address correspondence to Haiyan Chu, **hychu@issas.ac.cn**.

Di Wu and Yuying Ma contributed equally to this work.

Table S1 The nutrient properties (carbon, nitrogen, phosphorus, and potassium) of different organic matters (wheat straw, pig manure, and cow manure).

	**Organic Matters**		
**Annual Addition**	**Wheat Straw**	**Pig Manure**	**Cow Manure**
**(Fresh Base) (kg/hm^2)**	7500	15000	30000
**Average Water Content (%)**	33	68	78
**Organic Carbon Content (Dry Base) (g·kg^{-1})**	410	431	347
**Total Nitrogen Content (Dry Base) (g·kg^{-1})**	5.5	17.0	7.9
**Total Phosphorus Content (Dry Base) (g·kg^{-1})**	3.2	9.4	4.3
**Total Potassium Content (Dry Base) (g·kg^{-1})**	9.7	10.5	9.1

Table S2 Comparison on physiochemical variables among different treatments in three habitats. Data are means ± SD in parentheses and different letters in a column indicate significant differences (Duncan's test, P < 0.05). Each physiochemical property is measured from its respective habitat.

Habitat	Variable	Control	NPK	NPK+CM	NPK+PM	NPK+WS
**root endosphere**	TP / g kg^{-1}	0.366 (0.053)d	0.565 (0.038)c	1.04 (0.07)b	1.42 (0.10)a	0.579 (0.042)c
	TC / g kg^{-1}	402 (7)a	380 (6)b	386 (11)ab	380 (22)b	382 (8)b
	TN / g kg^{-1}	6.63 (0.77)ab	5.13 (1.90)ab	6.98 (1.76)ab	7.33 (1.41)a	4.66 (0.63)b
	TK / g kg^{-1}	4.32 (1.04)b	4.45 (1.02)b	6.59 (0.29)a	6.85 (0.67)a	5.79 (0.71)a
	C:N	61.2 (5.8)abc	80.9 (25.1)ab	57.7 (13.4)bc	52.9 (7.6)c	83.1 (11.8)a
	Ca / g kg^{-1}	2.05 (0.11)b	1.99 (0.20)b	2.58 (0.34)a	2.52 (0.22)a	1.74 (0.16)b
	Mg / g kg^{-1}	0.848 (0.135)b	1.02 (0.11)ab	0.998 (0.169)ab	1.17 (0.17)a	0.912 (0.090)b
	Na / g kg^{-1}	0.951 (0.108)b	0.886 (0.097)b	1.00 (0.13)b	1.42 (0.27)a	0.782 (0.202)b
	Fe / g kg^{-1}	2.56 (0.66)b	3.79 (0.48)ab	2.85 (0.89)ab	3.99 (1.25)a	3.18 (0.22)ab
	Mn / mg kg^{-1}	67.5 (10.4)b	150 (35)a	67.5 (11.9)b	137 (55)a	146 (13)a
	Zn / mg kg^{-1}	25.0 (4.1)b	16.3 (2.5)c	14.8 (2.1)c	55.0 (8.7)a	15.0 (1.8)c
**rhizosphere soil**	Moisture (%)	13.2 (3.2)bc	10.2 (2.4)c	20.9 (4.2)a	15.3 (2.6)b	15.0 (2.2)bc
	pH	7.15 (0.07)a	5.41 (0.14)c	7.06 (0.32)a	6.34 (0.14)b	5.52 (0.15)c
	DOC / mg kg^{-1}	47.3 (6.6)d	86.6 (14.9)c	134 (15)a	108 (19)bc	114 (10.7)ab
	DON / mg kg^{-1}	0.820 (0.485)c	5.63 (1.91)b	6.86 (2.46)ab	6.95 (3.72)ab	10.7 (3.1)a
	NO_3^--N / mg kg^{-1}	8.96 (1.74)b	18.9 (6.6)ab	35.4 (23.6)a	39.1 (15.3)a	8.18 (3.07)b
	NH_4^+-N / mg kg^{-1}	4.79 (0.26)a	6.01 (0.90)a	5.58 (0.58)a	5.86 (0.51)a	5.88 (3.17)a
	AP / mg kg^{-1}	2.37 (0.35)d	33.2 (6.3)c	78.9 (7.8)b	165 (36)a	27.4 (4.6)cd
	AK / mg kg^{-1}	129 (7)c	184 (64)c	825 (167)a	394 (53)b	347 (54)b
	TP / g kg^{-1}	0.268 (0.017)d	0.510 (0.071)c	0.915 (0.068)b	1.27 (0.07)a	0.473 (0.040)c
	TC / g kg^{-1}	8.91 (0.29)d	11.5 (0.7)cd	32.5 (7.2)a	17.3 (0.8)b	15.7 (1.3)bc
	TN / g kg^{-1}	0.848 (0.052)e	1.31 (0.12)d	2.82 (0.24)a	1.84 (0.14)b	1.59 (0.08)c
	TK / g kg^{-1}	13.9 (0.2)ab	12.6 (1.5)b	15.2 (1.1)a	14.2 (0.3)a	14.3 (0.4)a
	C:N	10.5 (0.5)ab	8.79 (0.44)c	11.4 (1.7)a	9.44 (1.01)bc	9.90 (0.53)bc
	Ca / g kg^{-1}	6.47 (1.07)a	5.57 (1.51)ab	6.79 (0.16)a	6.02 (1.11)ab	4.78 (0.52)b
	Mg / g kg^{-1}	5.85 (0.29)a	5.29 (0.08)c	5.64 (0.16)ab	5.45 (0.30)bc	5.25 (0.17)c
	Na / g kg^{-1}	10.7 (0.5)a	10.1 (0.1)bc	9.67 (0.40)c	9.86 (0.18)c	10.4 (0.2)ab
	Fe / g kg^{-1}	23.0 (1.5)a	23.0 (0.3)a	21.6 (0.7)a	21.7 (1.2)a	23.0 (0.8)a
	Mn / g kg^{-1}	372 (51)a	370 (55)a	361 (31)a	373 (51)a	394 (23)a
	Zn / mg kg^{-1}	44.1 (2.7)bc	42.3 (1.5)c	54.4 (1.4)b	107 (15)a	42.0 (0.9)c
	Moisture (%)	19.7 (2.3)b	18.3 (0.3)b	23.9 (2.1)a	19.1 (1.1)b	20.0 (1.0)b
	pH	6.98 (0.02)a	5.3 (0.3)c	7.2 (0.1)a	6.51 (0.22)b	5.21 (0.10)c
	DOC / mg kg^{-1}	48.4 (4.2)b	55.3 (4.5)b	90.4 (12.0)a	80.5 (9.7)a	87.3 (8.7)a
	DON / mg kg^{-1}	1.01 (0.97)b	2.13 (0.42)b	1.34 (0.85)b	1.89 (0.94)b	5.79 (3.66)a
	NO_3^--N / mg kg^{-1}	10.5 (1.0)b	31.3 (18.7)a	17.2 (2.0)ab	16.3 (1.4)ab	29.0 (9.5)a

	NH_4^+-N / mg kg^{-1}	4.81 (0.46)a	9.74 (8.38)a	5.25 (0.03)a	4.91 (0.13)a	6.65 (1.78)a
	AP / mg kg^{-1}	1.80 (0.79)d	24.7 (2.4)c	87.3 (3.3)b	165 (5)a	26.7 (3.1)c
	AK / mg kg^{-1}	125 (5)c	138 (11)c	547 (53)a	189 (9)b	189 (16)b
**bulk**	TP / g kg^{-1}	0.263 (0.017)d	0.410 (0.022)c	0.860 (0.061)b	1.07 (0.06)a	0.432 (0.022)c
**soil**	TC / g kg^{-1}	7.87 (0.56)c	9.30 (0.32)c	21.2 (1.7)a	14.4 (0.6)b	13.0 (1.2)b
	TN / g kg^{-1}	0.863 (0.050)e	1.10 (0.02)d	2.10 (0.09)a	1.50 (0.03)b	1.36 (0.10)c
	TK / g kg^{-1}	13.8 (0.3)c	14.3 (0.25)b	15.3 (0.3)a	14.1 (0.2)bc	14.1 (0.3)bc
	C:N	9.16 (0.97)bc	8.46 (0.46)c	10.1 (0.4)a	9.62 (0.41)ab	9.57 (0.34)ab
	Ca / g kg^{-1}	5.06 (0.75)bc	4.61 (0.34)c	6.90 (0.77)ab	7.80 (2.32)a	5.33 (1.32)bc
	Mg / g kg^{-1}	5.72 (0.36)a	5.18 (0.24)b	5.67 (0.32)a	5.42 (0.22)ab	5.30 (0.22)ab
	Na / g kg^{-1}	10.8 (0.5)a	10.4 (0.3)a	10.2 (0.3)a	10.4 (0.5)a	10.8 (0.4)a
	Fe / g kg^{-1}	23.3 (1.5)a	22.9 (0.8)a	22.2 (1.1)a	22.3 (1.3)a	23.3 (0.7)a
	Mn / g kg^{-1}	402.1 (41.7)a	386 (24)a	407 (33)a	400 (74)a	414 (62)a
	Zn / mg kg^{-1}	46.4 (5.1)c	43.0 (2.1)c	53.4 (3.8)b	84.8 (7.2)a	43 (2)c

Note: Moisture; AP, available phosphorus; AK, available potassium; TC, total carbon; TN, total nitrogen; TP, total phosphorus; TK, total potassium; C:N, total carbon / total nitrogen; DOC, dissolved organic carbon; NO_3^--N nitrate; NH_4^+-N, ammonium; DON, dissolved organic nitrogen; Ca, calcium; Mg, magnesium; Na, sodium; Fe, iron; Mn, manganese and Zn, zinc.

Table S3 Analysis of similarities (ANOSIM) of fungal community composition among treatments in three habitats (permutations = 999).

	**R**	***P***
Treatment	0.551	0.001
Habitat	0.461	0.001

Table S4 The pairwise Adonis comparison analysis of fungal community composition among treatments in three habitats (permutations = 999).

**Pair treatment**	**root endosphere**		**rhizosphere soil**		**bulk soil**	
	$\mathbf{R^2}$	***P***	$\mathbf{R^2}$	***P***	$\mathbf{R^2}$	***P***
Control vs NPK+PM	0.488	0.034	0.649	0.023	0.556	0.026
Control vs NPK+WS	0.331	0.034	0.384	0.033	0.411	0.026
Control vs NPK+CM	0.303	0.03	0.348	0.028	0.343	0.03
Control vs NPK	0.286	0.031	0.38	0.037	0.364	0.027
NPK+PM vs NPK+WS	0.59	0.035	0.675	0.036	0.832	0.034
NPK+PM vs NPK+CM	0.494	0.029	0.583	0.021	0.694	0.03
NPK+PM vs NPK	0.535	0.038	0.751	0.03	0.834	0.029
NPK+WS vs NPK+CM	0.347	0.027	0.387	0.032	0.59	0.036
NPK+WS vs NPK	0.188	0.124	0.36	0.025	0.5	0.032
NPK+CM vs NPK	0.31	0.039	0.445	0.036	0.569	0.025

Table S5 Comparison on relative abundances of fungi at phyla level in three habitats. Data are means ± SD in parentheses and different letters in a column indicate significant differences (Duncan's test, $P < 0.05$).

Habitat	Phyla	Control	NPK	NPK+CM	NPK+PM	NPK+WS
**root endosphere**	Ascomycota	41.2 (29.3)	46.4 (24.6)	58.3 (24.3)	96.9 (3.6)	45.7 (30.9)
	Basidiomycota	47.1 (28.1)	51.7 (24.6)	36.5 (23.7)	2.30 (3.09)	53.1 (31.8)
	Mortierellomycota	0.034 (0.018)	0.091 (0.024)	0.274 (0.508)	0.037 (0.034)	0.119 (0.092)
**rhizosphere soil**	Ascomycota	71.2 (16.8)ab	62.3 (11.8)ab	50.7 (5.316)a	87.1 (5.9) b	73.5 (13.2)ab
	Basidiomycota	18.1 (18.2)	16.6 (8.9)	22.8 (16.6)	2.1 (2.3)	17.8 (13.9)
	Mortierellomycota	5.75 (3.46)	17.8 (7.4)	11.6 (13.7)	8.01 (5.60)	5.68 (3.78)
**bulk soil**	Ascomycota	81.7 (12.6)bc	72.8 (9.3) bc	42.6 (16.6)a	91.1 (6.2) c	64.7 (11.1)ab
	Basidiomycota	9.30 (7.79)	3.46 (0.57)	8.97 (8.81)	0.2 (0.1)	9.64 (2.92)
	Mortierellomycota	5.19 (2.54)a	17.8 (9.0)ab	27.0 (16.3) b	6.41 (3.94)ab	22.1 (10.0)ab

Table S6 Comparison on relative abundances of fungi at class level in three habitats (relative abundance > 0.5%). Data are means ± SD in parentheses and different letters in a column indicate significant differences (Duncan's test, $P < 0.05$).

Habitat	Class	Control	NPK	NPK+CM	NPK+PM	NPK+WS
	Sordariomycetes	28.5 (28.2)a	18.9 (18.4)a	38.7 (27.7)ab	87.7 (8.4) b	20.6 (25.8)a
	Agaricomycetes	47.0 (28.1)	51.0 (24.8)	35.6 (23.3)	2.02 (2.69)	51.6 (31.6)
	Pezizomycetes	0.031 (0.027)ab	0.007 (0.014)a	0.022 (0.044)ab	0.126 (0.112) b	0a
	Mortierellomycetes	0.034 (0.018)	0.091 (0.024)	0.274 (0.508)	0.037 (0.034)	0.119 (0.092)
	Eurotiomycetes	0.853 (0.802)	0.641 (0.545)	4.75 (5.01)	1.44 (1.99)	2.77 (2.55)
	Leotiomycetes	4.57 (3.24)	11.9 (8.8)	6.18 (3.93)	2.85 (1.22)	18.8 (15.2)
	Dothideomycetes	5.19 (3.82)	13.8 (17.9)	5.41 (4.53)	2.76 (2.95)	2.43 (2.69)
**root**	Tremellomycetes	0.008 (0.010)	0.058 (0.058)	0.073 (0.076)	0.037 (0.057)	0.191 (0.156)
**endosphere**	Pucciniomycetes	0.108 (0.123)a	0.391 (0.319)ab	0a	0a	0.948 (0.469) b
	Microbotryomycetes	0.012 (0.009)	0.086 (0.090)	0.084 (0.076)	0.057 (0.062)	0.189 (0.208)
	Lecanoromycetes	0	0	0.008 (0.016)	0	0
	Malasseziomycetes	0.014 (0.028)a	0.047 (0.044)ab	0.588 (0.522) b	0.073 (0.094)ab	0.113 (0.159)ab
	Ustilaginomycetes	0.003 (0.007)	0	0	0	0.003 (0.007)
	Exobasidiomycetes	0	0	0.002 (0.005)	0	0.017 (0.021)
	Cystobasidiomycetes	0.051 (0.074)	0.099 (0.183)	0.029 (0.035)	0.005 (0.005)	0.029 (0.046)
	Archaeorhizomycetes	0.002 (0.005)	0.015 (0.027)	0	0	0
	Orbiliomycetes	0.001 (0.002)	0	0.003 (0.007)	0	0.006 (0.012)
	Wallemiomycetes	0	0.006 (0.012)	0.054 (0.043)	0.109 (0.188)	0.028 (0.027)
	Sordariomycetes	31.6 (15.8)bc	19.8 (4.3)ab	24.8 (17.1)ac	2.15 (1.29)a	48.6 (7.8)c
	Agaricomycetes	14.3 (18.4)	12.2 (10.0)	22.3 (16.5)	1.95 (2.21)	15.5 (14.3)
	Pezizomycetes	0.153 (0.305)a	0.005 (0.009)a	0.091 (0.095)a	65.9 (6.07) b	0.002 (0.005)a
	Mortierellomycetes	5.75 (3.46)	17.8 (7.4)	11.6 (13.8)	8.01 (5.60)	5.68 (3.78)
	Eurotiomycetes	0.985 (1.438)a	16.2 (10.7)b	2.45 (3.79)a	0.021 (0.042)a	5.67 (2.94)ab
	Leotiomycetes	9.04 (14.1)	6.34 (3.92)	2.63 (1.34)	0.362 (0.386)	4.52 (1.94)
	Dothideomycetes	3.64 (1.05)	6.92 (1.65)	11.3 (10.1)	2.23 (2.30)	6.26 (2.66)
	Tremellomycetes	1.78 (0.35) b	2.33 (0.83) b	0.171 (0.079)a	0.122 (0.051)a	0.575 (0.063)a
**rhizosphere**	Pucciniomycetes	0.799 (1.410)	1.56 (1.42)	0	0.043 (0.030)	1.49 (1.17)
**soil**	Microbotryomycetes	0.779 (0.265) b	0.508 (0.060) b	0.105 (0.076)a	0.008 (0.010)a	0.156 (0.114)a
	Lecanoromycetes	0	0.069 (0.128)	0	0	0
	Spizellomycetes	0.464 (0.723)	0.029 (0.058)	0.031 (0.063)	0	0
	Ustilaginomycetes	0.108 (0.196)	0	0	0	0
	Exobasidiomycetes	0.119 (0.036)ab	0.057 (0.022)ab	0.150 (0.143) b	0a	0.030 (0.011)ab
	Cystobasidiomycetes	0.147 (0.102) b	0.027 (0.024)a	0.012 (0.013)a	0a	0a
	Archaeorhizomycetes	0.006 (0.012)	0	0	0	0
	Orbiliomycetes	0.070 (0.140)	0.119 (0.080)	0.007 (0.014)	0	0.005 (0.009)
	Rhizophlyctidomycetes	0	0.055 (0.065)	0.082 (0.163)	0	0

	Sordariomycetes	55.6 (21.2)c	26.3 (4.8)ab	33.8 (13.4)bc	2.50 (1.83)a	47.4 (8.0)bc
	Agaricomycetes	8.13 (7.44)	1.23 (1.11)	8.67 (8.90)	0.038 (0.036)	7.96 (3.85)
	Pezizomycetes	0.339 (0.099)a	0a	0.133 (0.139)a	74.4 (5.4)b	0a
	Mortierellomycetes	5.19 (2.54)a	17.8 (9.0)ab	27.0 (16.3)b	6.41 (3.94)ab	22.1 (10.0)ab
	Eurotiomycetes	5.32 (8.63)a	29.0 (11.8)b	0.104 (0.120)a	0.005 (0.009)a	5.24 (3.12)a
	Leotiomycetes	0.437 (0.383)	1.19 (1.15)	0.547 (0.418)	0.023 (0.028)	2.66 (2.95)
	Dothideomycetes	2.59 (2.19)	2.15 (0.85)	0.782 (0.305)	0.057 (0.064)	1.93 (2.21)
**bulk**	Tremellomycetes	0.292 (0.115)ab	1.37 (0.31)c	0.128 (0.098)a	0.157 (0.073)a	0.987 (0.762)bc
**soil**	Pucciniomycetes	0.147 (0.176)	0.535 (0.758)	0	0.007 (0.008)	0.683 (0.585)
	Microbotryomycetes	0.014 (0.018)	0.295 (0.559)	0	0	0.015 (0.014)
	Lecanoromycetes	0	1.51 (3.01)	0.064 (0.082)	0.058 (0.116)	0.040 (0.051)
	Spizellomycetes	0.553 (0.619)	0.043 (0.054)	0	0	0
	Ustilaginomycetes	0.571 (0.642)	0.022 (0.041)	0	0	0
	Exobasidiomycetes	0.026 (0.051)	0.009 (0.019)	0.005 (0.009)	0	0
	Cystobasidiomycetes	0.010 (0.021)	0	0	0	0
	Archaeorhizomycetes	0.373 (0.667)	0	0	0	0
	Orbiliomycetes	0.111 (0.148)	0.045 (0.067)	0	0	0.006 (0.012)
	Rhizophlyctidomycetes	0.076 (0.057)	0.128 (0.170)	0	0	0.024 (0.030)

Table S7 Results of the multiple regression analysis on matrices analysis (MRM) in three habitats.

root endosphere $R^2 = 0.112$, $P < 0.001$			rhizosphere soil $R^2 = 0.335$, $P < 0.001$			bulk soil $R^2 = 0.452$, $P < 0.001$		
**Variable**	***b***	***P***	**Variable**	***b***	***P***	**Variable**	***b***	***P***
TP	0.086	<0.001	AP	0.120	<0.001	AP	0.219	<0.001
			pH	0.095	<0.001	pH	0.122	<0.001
			DOC	0.038	0.019	TK	0.074	<0.001
						Ca	-0.075	0.004

Note: TP: total phosphors; AP, available phosphorous; DOC, dissolved organic carbon; Fe, iron; TK, total potassium; Ca, calcium. Each physiochemical property is measured from its respective habitat. The variation (R^2) of ln (Bray–Curtis distance) that is explained by the remaining variables. The partial regression coefficients (*b*) and associated *P*-values of the second model are reported from permutation test (nperm = 9999).

Table S8 Correlations among the most abundant phyla and physiochemical variables in three habitats (* *P* < 0.05;** *P* < 0.01; *** *P* < 0.001).

Habitat	Variable	Ascomycota	Basidiomycota	Mortierellomycota	Unidentified
	C:N	-0.395	0.411	0.238	-0.019
	Ca	0.314	-0.019	-0.314	0.183
	Fe	0.225	-0.182	0.210	-0.341
	Mg	0.265	-0.293	0.113	-0.140
**root**	Mn	0.104	-0.066	0.334	-0.444
**endosphere**	Na	**0.461***	**-0.500***	-0.030	0.058
	TC	-0.207	0.119	-0.352	0.267
	TK	**0.488***	**-0.532***	-0.105	0.209
	TN	0.289	-0.319	-0.287	0.073
	TP	**0.518***	**-0.461***	-0.014	-0.044
	Zn	0.261	-0.311	-0.307	-0.068
	AK	-0.113	-0.146	-0.070	0.405
	AP	0.089	-0.422	0.155	0.059
	C:N	-0.149	0.115	**-0.451***	0.439
	Ca	-0.078	-0.183	0.247	0.225
	DOC	-0.117	0.029	-0.100	0.394
	DON	0.117	-0.213	0.058	-0.100
	Fe	-0.209	0.263	0.327	**-0.524***
	Mg	-0.345	0.142	0.062	0.272
	Mn	-0.059	-0.090	0.130	-0.221
**rhizosphere**	Moisture	-0.055	-0.089	-0.272	**0.476***
**soil**	Na	0.033	0.189	0.062	-0.250
	NH_4^+-N	-0.015	0.053	0.206	-0.198
	NO_3^--N	-0.081	-0.409	0.198	0.053
	pH	-0.219	0.134	-0.242	**0.576****
	TC	-0.141	-0.083	-0.106	0.412
	TK	-0.134	-0.064	-0.142	0.460*
	TN	-0.101	-0.169	-0.071	0.369
	TP	0.126	-0.432	0.119	0.088
	Zn	0.193	-0.358	-0.130	0.125
	AK	**-0.483***	0.039	0.437	0.333
	AP	0.062	**-0.523***	0.059	-0.025
	C:N	-0.194	-0.081	0.194	0.035
	Ca	-0.072	-0.180	-0.021	0.220
	DOC	**-0.486***	0.024	**0.568****	0.343
	DON	-0.102	0.133	0.310	-0.123

	Fe	-0.068	0.425	0.081	0.127
	Mg	-0.079	0.353	-0.120	0.250
	Mn	-0.082	0.066	-0.011	0.181
**bulk**	Moisture	-0.359	0.157	0.238	0.029
**soil**	Na	-0.086	0.423	0.063	0.173
	NH_4^+-N	**-0.546***	0.235	**0.618****	0.150
	NO_3^--N	**-0.480***	0.026	**0.702*****	0.235
	pH	-0.075	0.089	-0.202	0.278
	TC	-0.380	-0.139	0.432	0.272
	TK	**-0.645****	0.043	**0.649****	**0.558***
	TN	-0.387	-0.143	0.399	0.312
	TP	0.059	**-0.473***	0.028	-0.008
	Zn	0.205	-0.403	-0.281	0.056

Note: Moisture; AP, available phosphorus; AK, available potassium; TC, total carbon; TN, total nitrogen; TP, total phosphorus; TK, total potassium; C:N, total carbon / total nitrogen; DOC, dissolved organic carbon; NO_3^--N nitrate; NH_4^+-N, ammonium; DON, dissolved organic nitrogen; Ca, calcium; Mg, magnesium; Na, sodium; Fe, iron; Mn, manganese and Zn, zinc. Each physiochemical property is measured from its respective habitat.

Table S9 Correlations among the most abundant classes and physiochemical variables in three habitats (relative abundance >0.5%; * $P < 0.05$, ** $P < 0.01$, *** $P < 0.001$). Each physiochemical property is measured from its respective habitat.

**root endosphere**											
**Class**	**C:N**	**Ca**	**Fe**	**Mg**	**Mn**	**Na**	**TC**	**TK**	**TN**	**TP**	**Zn**
Sordariomycetes	-0.260	0.377	0.011	0.104	0.027	0.335	0.082	**0.556***	0.216	**0.488***	0.199
Agaricomycetes	0.418	-0.337	-0.212	-0.302	-0.091	**-0.511***	0.155	**-0.514***	-0.319	**-0.458***	-0.311
Unidentified	-0.144	0.366	-0.305	-0.028	**-0.500***	0.219	0.247	0.118	0.203	-0.160	0.054
Pezizomycetes	-0.224	0.447	0.132	0.204	0.040	0.339	0.184	0.099	0.250	0.066	**0.562***
Mortierellomycetes	0.238	-0.314	0.210	0.113	0.334	-0.030	-0.352	-0.105	-0.287	-0.014	-0.307
Eurotiomycetes	-0.049	0.053	-0.104	-0.097	0.029	-0.030	-0.126	0.440	0.019	0.304	-0.323
Leotiomycetes	0.426	**-0.569***	-0.002	-0.138	0.253	-0.326	-0.251	-0.130	-0.453	-0.119	-0.453
Dothideomycetes	0.026	0.160	0.116	0.071	-0.199	-0.154	0.027	-0.360	0.000	-0.326	0.126
Tremellomycetes	0.183	-0.238	0.246	0.083	0.419	-0.074	-0.425	0.155	-0.223	0.186	-0.285
Pucciniomycetes	**0.571***	**-0.698****	0.141	-0.200	0.430	**-0.571***	-0.124	**-0.520***	**-0.563***	**-0.512***	-0.266
Microbotryomycetes	0.020	-0.019	0.349	0.283	0.270	0.005	**-0.474***	0.236	-0.089	0.267	-0.257
Lecanoromycetes	0.086	0.344	0.258	0.301	-0.043	0.215	-0.345	0.172	-0.086	0.129	0.022
Malasseziomycetes	-0.034	0.257	0.158	0.337	0.039	0.102	-0.344	**0.477***	0.017	0.438	-0.298
Ustilaginomycetes	0.063	-0.031	0.031	0.219	0.157	0.219	-0.031	0.031	-0.094	-0.188	0.157
Exobasidiomycetes	0.320	-0.086	0.022	0.084	0.100	-0.135	-0.221	0.233	-0.405	0.094	-0.232
Cystobasidiomycetes	0.318	-0.227	-0.008	-0.168	0.190	-0.402	0.072	-0.114	-0.300	-0.152	-0.061
Archaeorhizomycetes	0.362	-0.283	-0.115	-0.283	0.176	-0.300	0.120	-0.396	-0.312	-0.359	-0.145
Orbiliomycetes	0.095	-0.048	-0.429	-0.362	-0.143	-0.360	0.394	-0.146	-0.043	-0.022	-0.024
Wallemiomycetes	-0.104	0.337	0.275	0.389	0.033	0.235	**-0.510***	**0.539***	0.019	0.425	-0.179

									rhizosphere soil										
**Class**	**AK**	**AP**	**C:N**	**Ca**	**DOC**	**DON**	**Fe**	**Mg**	**Mn**	**Moisture**	**Na**	**NH_4^+-N**	**NO_3^--N**	**pH**	**TC**	**TK**	**TN**	**TP**	**Zn**
Sordariomycetes	-0.171	**-0.645****	-0.129	-0.111	0.017	0.182	0.167	-0.099	0.138	-0.075	**0.516***	-0.135	**-0.618****	-0.126	-0.262	-0.086	-0.271	**-0.608****	**-0.658****
Agaricomycetes	0.035	-0.192	0.263	-0.162	0.179	-0.077	0.205	0.128	-0.032	0.023	0.051	-0.098	-0.244	0.156	0.138	0.102	0.029	-0.224	-0.202
Unidentified	0.097	0.`045	0.242	**0.547***	-0.102	**-0.531***	-0.367	0.384	-0.038	0.289	-0.259	**-0.477***	0.135	**0.604****	-0.002	0.320	0.066	0.129	0.296
Pezizomycetes	**0.491***	**0.678****	0.252	0.276	0.328	0.141	-0.427	0.059	-0.023	0.273	**-0.487***	0.043	**0.595****	0.253	**0.471***	0.239	**0.513***	**0.693*****	**0.724*****
Mortierellomycetes	-0.070	0.155	**-0.451***	0.247	-0.100	0.058	0.327	0.062	0.130	-0.272	0.062	0.206	0.198	-0.242	-0.106	-0.142	-0.071	0.119	-0.130
Eurotiomycetes	-0.227	-0.262	-0.427	-0.244	0.042	0.309	0.345	**-0.487***	0.014	-0.463*	0.105	0.202	-0.289	**-0.652****	-0.171	-0.238	-0.198	-0.271	**-0.561***
Leotiomycetes	-0.386	-0.516*	-0.353	-0.138	-0.229	-0.072	0.405	-0.149	-0.017	-0.320	0.319	0.020	-0.412	-0.329	-0.320	-0.135	-0.374	**-0.565****	**-0.676****
Dothideomycetes	0.203	0.000	0.062	-0.092	0.379	0.245	0.014	-0.219	-0.135	-0.148	-0.230	-0.071	-0.197	-0.167	0.189	0.155	0.147	0.008	-0.263
Tremellomycetes	**-0.850*****	**-0.742*****	**-0.649****	-0.122	**-0.630****	-0.400	**0.490***	-0.071	0.065	**-0.593****	**0.611****	-0.201	**-0.522***	-0.312	**-0.836*****	**-0.591****	**-0.867*****	**-0.766*****	**-0.671****
Pucciniomycetes	**-0.579****	**-0.562****	**-0.496***	-0.450*	-0.285	-0.002	0.414	**-0.446***	0.147	**-0.476***	**0.539***	-0.053	**-0.576****	**-0.665*****	**-0.624****	**-0.527***	**-0.608****	**-0.507***	**-0.640****
Microbotryomycetes	**-0.743*****	**-0.767*****	**-0.446***	0.047	**-0.644****	**-0.498***	0.391	0.187	-0.023	**-0.481***	**0.589****	-0.374	**-0.562****	0.011	**-0.749*****	-0.382	**-0.794*****	**-0.799*****	**-0.634****
Lecanoromycetes	-0.172	0.004	-0.400	-0.063	-0.290	-0.007	0.235	-0.208	-0.033	-0.336	-0.141	0.127	0.199	-0.378	-0.232	-0.033	-0.232	-0.064	**-0.492***
Spizellomycetes	-0.494*	**-0.506***	-0.409	0.302	**-0.532***	**-0.447***	0.034	0.395	-0.172	-0.140	**0.451***	-0.253	-0.160	0.256	**-0.480***	-0.381	**-0.462***	**-0.552***	-0.151
Ustilaginomycetes	-0.345	**-0.518***	-0.293	0.186	-0.430	-0.430	-0.221	0.232	-0.309	-0.046	0.257	-0.408	-0.229	0.289	**-0.488***	-0.257	-0.430	**-0.488***	-0.273
Exobasidiomycetes	-0.310	**-0.550***	-0.032	0.310	-0.263	**-0.444***	0.062	0.417	-0.048	-0.070	0.249	-0.439	-0.351	0.408	-0.304	-0.089	-0.317	**-0.540***	-0.189
Cystobasidiomycetes	**-0.554***	**-0.464***	-0.323	0.220	**-0.453***	-0.413	0.368	0.409	0.038	**-0.538***	0.374	-0.214	-0.226	0.209	**-0.458***	**-0.453***	**-0.482***	**-0.491***	-0.067
Archaeorhizomycetes	-0.378	-0.259	-0.298	0.378	-0.378	-0.298	0.378	0.378	0.060	-0.299	0.378	-0.259	-0.259	0.259	-0.338	-0.259	-0.378	-0.338	0.060
Orbiliomycetes	-0.410	-0.270	**-0.600****	0.028	-0.319	-0.134	0.344	-0.152	0.079	**-0.472***	0.139	0.072	0.106	-0.407	-0.379	-0.362	-0.369	-0.301	**-0.450***
Rhizophlyctidomycetes	-0.046	0.048	-0.111	0.034	-0.036	-0.065	0.051	-0.219	-0.116	-0.140	-0.247	-0.048	0.063	-0.295	0.036	0.085	0.036	0.075	-0.103

bulk soil																			
**Class**	**AK**	**AP**	**C:N**	**Ca**	**DOC**	**DON**	**Fe**	**Mg**	**Mn**	**Moisture**	**Na**	**NH_4^+-N**	**NO_3^--N**	**pH**	**TC**	**TK**	**TN**	**TP**	**Zn**
Sordariomycetes	-0.223	**-0.643****	0.059	-0.341	-0.257	0.114	0.197	0.200	-0.050	0.355	0.177	0.211	-0.244	0.096	-0.350	-0.142	-0.429	**-0.664****	**-0.578****
Agaricomycetes	0.122	**-0.451***	0.036	-0.099	0.066	0.038	0.323	0.393	0.111	0.269	0.316	0.153	-0.042	0.235	-0.063	0.058	-0.079	-0.430	-0.309
Unidentified	0.062	0.030	0.074	-0.024	-0.078	**-0.609****	-0.310	0.099	-0.152	0.095	-0.173	-0.155	-0.281	0.392	0.068	0.234	0.095	0.035	0.243
Pezizomycetes	-0.014	0.353	0.134	**0.479***	-0.123	-0.423	-0.189	0.189	0.192	-0.091	-0.170	**-0.741***	**-0.637***	**0.467***	0.066	-0.267	0.140	0.379	**0.677***
Mortierellomycetes	0.437	0.059	0.194	-0.021	**0.568****	0.310	0.081	-0.120	-0.011	0.238	0.063	**0.618****	**0.702*****	-0.202	0.432	**0.649****	0.399	0.028	-0.281
Eurotiomycetes	**-0.524***	**-0.659****	**-0.620****	**-0.559***	**-0.450***	0.111	**0.447***	-0.323	-0.026	**-0.479***	0.218	0.383	0.312	**-0.571****	**-0.641****	-0.191	**-0.676****	**-0.636****	**-0.763*****
Leotiomycetes	-0.011	-0.425	-0.117	-0.325	0.058	0.193	0.396	-0.227	0.153	0.092	0.158	**0.667****	0.429	-0.406	-0.148	-0.004	-0.212	-0.328	**-0.585****
Dothideomycetes	-0.375	**-0.704*****	**-0.484***	-0.442	-0.367	-0.030	**0.472***	0.090	-0.111	-0.332	**0.456***	0.244	0.155	-0.169	**-0.499***	0.008	**-0.517***	**-0.684*****	**-0.597****
Tremellomycetes	-0.337	**-0.455***	**-0.542***	-0.429	-0.121	0.252	**0.516***	-0.316	-0.032	-0.409	0.263	0.394	0.436	**-0.692***	**-0.471***	-0.079	**-0.488***	**-0.446*****	**-0.723*****
Pucciniomycetes	-0.379	-0.417	-0.122	**-0.502***	-0.152	**0.698***	0.252	-0.341	-0.197	-0.127	0.342	**0.628***	**0.472***	**-0.773***	-0.369	-0.171	**-0.477***	**-0.453***	**-0.664***
Microbotryomycetes	-0.363	**-0.575****	-0.412	-0.412	-0.250	0.299	**0.474***	0.017	-0.086	-0.380	**0.686*****	0.410	0.371	-0.429	-0.431	-0.233	-0.427	-0.487*	-0.395
Lecanoromycetes	0.258	0.159	-0.030	-0.175	0.026	0.217	-0.189	-0.049	-0.206	-0.131	-0.053	0.187	0.198	-0.081	0.235	0.182	0.268	0.138	-0.021
Spizellomycetes	**-0.686*****	**-0.687*****	-0.422	-0.310	**-0.712*****	**-0.499***	0.184	0.252	-0.058	-0.287	0.156	**-0.515***	**-0.633****	0.259	**-0.768*****	-0.437	**-0.766*****	**-0.739*****	-0.231
Ustilaginomycetes	**-0.574****	**-0.518***	-0.405	-0.340	**-0.575****	**-0.486***	0.024	0.049	-0.069	-0.350	-0.014	-0.425	**-0.447***	0.119	**-0.628****	-0.277	**-0.625****	**-0.579****	-0.208
Exobasidiomycetes	-0.046	-0.220	-0.126	0.075	-0.114	-0.283	**0.566****	0.438	**0.453***	-0.152	0.348	-0.017	-0.167	0.215	-0.131	-0.170	-0.114	-0.111	0.131
Cystobasidiomycetes	-0.179	-0.299	-0.298	0.060	-0.219	-0.338	0.378	0.378	0.338	-0.378	0.378	-0.378	-0.338	0.139	-0.378	-0.339	-0.299	-0.259	0.139
Archaeorhizomycetes	-0.412	-0.403	0.052	-0.247	-0.439	-0.218	0.003	0.336	0.066	-0.063	0.306	-0.245	**-0.460***	0.172	**-0.457***	**-0.522***	**-0.494***	-0.436	0.111
Orbiliomycetes	**-0.535***	**-0.569****	**-0.610****	-0.232	-0.378	-0.143	0.409	-0.101	0.134	**-0.464***	0.248	0.070	0.172	-0.204	**-0.569****	-0.080	**-0.532***	**-0.534***	-0.407
Rhizophlyctidomycetes	**-0.597****	**-0.648****	-0.316	**-0.476***	**-0.535***	-0.142	0.328	-0.111	-0.200	-0.249	0.268	-0.005	-0.068	-0.124	**-0.540***	-0.350	**-0.670****	**-0.584****	**-0.519***

Note: AP, available phosphorus; AK, available potassium; TC, total carbon; TN, total nitrogen; TP, total phosphorus; TK, total potassium; C:N, total carbon / total nitrogen; DOC, dissolved organic carbon; NO_3^--N nitrate; NH_4^+-N, ammonium; DON, dissolved organic nitrogen; Ca, calcium; Mg, magnesium; Na, sodium; Fe, iron; Mn, manganese and Zn, zinc.

Table S10 Details about some taxa had significant difference of top 50 abundant ASVs maong treatments in each habitat. Correlations among the relative abundance of these ASVs and physiochemical variables in three habitats (* $P < 0.05$;** $P < 0.01$; *** $P < 0.001$). Each physiochemical property is measured from its respective habitat.

**Habitat**	**ASV**	**ID**	**Qiime2**	**BLASTn**	**Identity**	**TP**	**Zn**
**root endosphere**	ASV1067	d0a957367c04253f1ac0431205b345f0	Xylariales	*Microdochium*	92%	-0.597**	
	ASV1069	c3495ddecf2fba082a67269b30b03cad	Sordariomycetes	*Arthropsis truncata*	82.51%	0.735***	
	ASV1074	62bff820521975c7033830b7b80396d3	Helotiales	*Hyalodendriella betulae*	81.15%	0.516*	
	ASV1322	a91effc1fecf083601962b306a6cea5a	*Unidentified*	*Basidiobolus*	98.21%		
	ASV219	ec5bf20a414e5da394f9a4b776c83035	*Exophiala*	*Exophiala*	98.93%		-0.611**
	ASV340	74946621f1d772847107b7a41c803eb8	Agaricomycetes	*Subulicystidium*	85.95%		-0.689**
	ASV445	d005304dc9498cd68c4b02510ff5932e	*Mortierella*	*Mortierella*	98.54%		
	ASV813	cabc7d7f59a6b311e516f26a45786a15	Strophariaceae	*Deconica*	94.85%	-0.610**	-0.489*

**Habitat**	**ASV**	**ID**	**Qiime2**	**BLASTn**	**Identity**	**AP**	**pH**	**Zn**
**rhizosphere soil**	ASV1009	68adf76c449f42b1e3c4c7022167768e	Hypocreales	*Fusarium solani*	97.17%			
	ASV1074	62bff820521975c7033830b7b80396d3	Helotiales	*Hyalodendriella betulae*	81.15%			
	ASV1300	addad095e5c0ea04b2d27bcc07ee1de6	Pyronemataceae	*Tricharina*	89.34%	0.589**		0.642**
	ASV1318	23a39cdada96f9cd253b6f9f1c07addf	Sordariales	*Echria gigantospora*	98.19%		-0.457*	
	ASV1322	a91effc1fecf083601962b306a6cea5a	Unidentified	*Basidiobolus*	98.21%	0.535*		
	ASV1467	8e28720e0fe8b7ba4f6a82ba1021d116	*Myrmecridium*	*Myrmecridium*	94.44%	-0.533*	0.683**	
	ASV1590	3cb753e9f440a12f20059fc54655c084	*Mortierella*	*Linnemannia*	97.55%	0.762***		0.783***
	ASV1591	ced7bdc5b89b6f62aaa8f4777d01fd8a	Chaetothyriales	*Chaetothyrium*	88.18%		-0.733***	-0.516*
	ASV219	ec5bf20a414e5da394f9a4b776c83035	*Exophiala*	*Exophiala*	98.93%		-0.737***	-0.642**
	ASV223	d5e8835df78b01881ed85ad23995b2b9	Sordariales	*Chaetomium*	96.75%		-0.468*	-0.449*

	ASV309	5a63e83bb6b5f3d4c658e61b172e41f1	*Marasmius*	*Marasmius*	88.47%	-0.547*	0.489*	
	ASV340	74946621f1d772847107b7a41c803eb8	Agaricomycetes	*Subulicystidium*	85.95%		0.520*	
	ASV445	d005304dc9498cd68c4b02510ff5932e	*Mortierella*	*Mortierella*	98.54%	-0.478*	-0.607**	-0.751***
	ASV600	762d98bdefb46d1f50b7ce84a136d6c8	*Knufia*	*Knufia*	89.68%		-0.701**	-0.512*
	ASV727	43e89bb9af853dd4b4abb71175c6f634	Xylariales	*Nothodactylaria*	88.02%	-0.807***		-0.664**
	ASV759	c5ed5cd3a42c528bb163c46220e3cb13	Ascomycota	*Eleutherascus*	84.07%	0.532*		0.599**
	ASV1004	6ec1f56cf30e408136093bdff9d843e1	*Mortierella*	*Linnemannia*	97.06%	0.750***	0.582**	0.727***
	ASV1256	db9af590f13ca77c4902108783f708c1	Pleosporales	*Alternaria alstroemeriae*	100%	-0.694***		-0.627**
	ASV1300	addad095e5c0ea04b2d27bcc07ee1de6	Pyronemataceae	*Tricharina*	89.34%			0.622**
	ASV1590	3cb753e9f440a12f20059fc54655c084	*Mortierella*	*Linnemannia*	97.55%	0.730***	0.602**	0.674**
	ASV1591	ced7bdc5b89b6f62aaa8f4777d01fd8a	Chaetothyriales	*Chaetothyrium*	88.18%		-0.574**	-0.595**
**bulk**	ASV219	ec5bf20a414e5da394f9a4b776c83035	*Exophiala*	*Exophiala*	98.93%		-0.629**	-0.566**
**soil**	ASV223	d5e8835df78b01881ed85ad23995b2b9	Sordariales	*Chaetomium*	96.75%		-0.597**	
	ASV445	d005304dc9498cd68c4b02510ff5932e	*Mortierella*	*Mortierella*	98.54%	-0.461*	-0.610**	-0.734***
	ASV471	45ca57c728b91c0ed21958bc99e43a42	Sordariales	*Apodus deciduus*	99.59%			-0.638**
	ASV727	43e89bb9af853dd4b4abb71175c6f634	Xylariales	*Nothodactylaria*	88.02%	-0.684***		-0.633**
	ASV759	c5ed5cd3a42c528bb163c46220e3cb13	Ascomycota	*Eleutherascus*	84.07%			0.674**
	ASV920	8f7b73a6de6f79e6458fcfa68ea00fb8	Ascomycota	*Condenascus tortuosus*	100%	-0.601**		-0.672**
	ASV949	3d7c15e19a1ce7b3c7605bbdc350fbc5	Ascomycota	*Chaetothyrium*	88.18%	-0.490*		-0.453*
	ASV99	566f04e4a32c05b558704d21007ef0f3	Nectriaceae	*Fusarium guttiforme*	99.59%	-0.539*	-0.626**	-0.808***
	ASV994	b697550f05e2cc7f87c8caf010f75d2b	Sordariales	*Coniochaeta*	94.67%		0.646**	

Table S11 Relationships between physiochemical variables and the co-occurrence network characters in three habitats. Data are fitted using linear regression.

Habitat	Variable	Average degree			Edges			Nodes		
		Estimate	R^2	*P*	Estimate	R^2	*P*	Estimate	R^2	*P*
**root endosphere**	TP	-3.800	**0.456**	0.002	-119.09	**0.231**	0.037	-4.906	0.035	0.445
	Zn	-0.071	**0.252**	0.029	-2.482	0.158	0.092	-0.195	0.0867	0.221
**rhizosphere soil**	AP	-0.028	**0.745**	0.000	-1.450	**0.776**	0.000	0.573	**0.573**	0.000
	TP	-4.410	**0.708**	0.000	-231.190	**0.742**	0.000	-32.442	**0.521**	0.000
	Zn	-0.065	**0.759**	0.000	-3.497	**0.843**	0.000	-0.541	**0.718**	0.000
	pH	0.443	0.031	0.460	-17.100	0.017	0.579	-8.850	0.167	0.074
**bulk soil**	AP	-0.039	**0.730**	0.000	-1.803	**0.700**	0.000	-0.235	**0.742**	0.000
	TP	-6.822	**0.597**	0.000	-320.05	**0.598**	0.000	-41.676	**0.631**	0.000
	Zn	-0.151	**0.818**	0.000	-6.784	**0.751**	0.000	-0.886	**0.798**	0.000
	pH	-1.649	**0.261**	0.021	-84.78	**0.314**	0.010	-8.402	0.192	0.054

Note: AP, available phosphorus; TP, total phosphorus; Zn, zinc. Each physiochemical property is measured from its respective habitat.

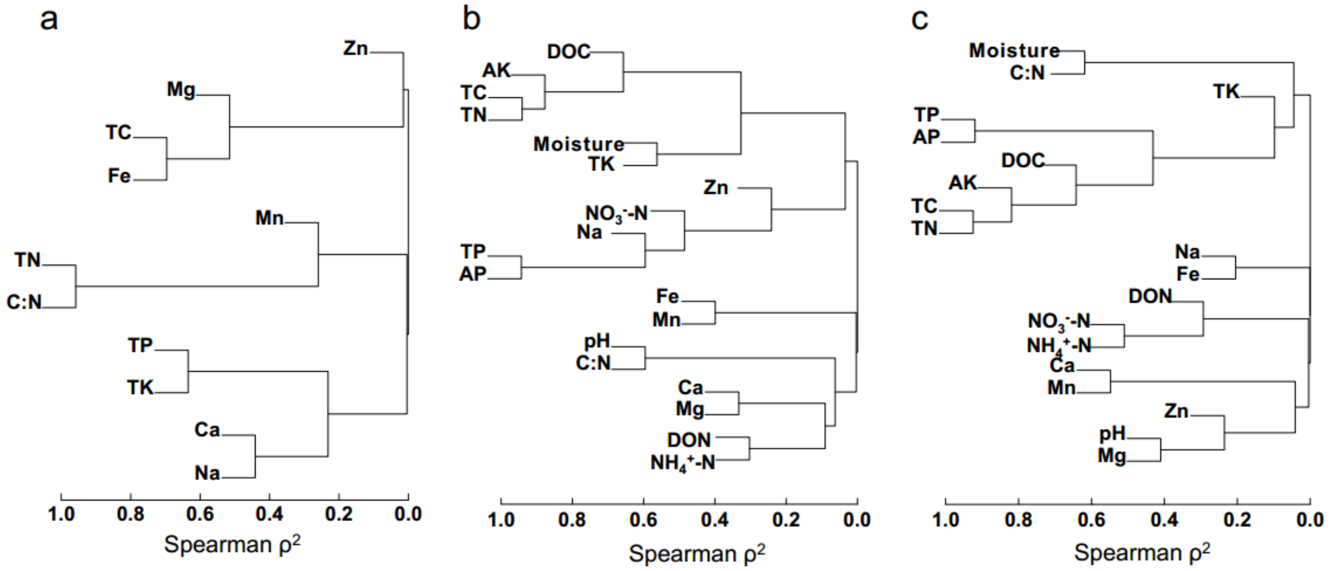

Fig. S1 Cluster analysis of the measured physiochemical variables in three habitats. Moisture; AP, available phosphorus; AK, available potassium; TC, total carbon; TN, total nitrogen; TP, total phosphorus; TK, total potassium; C:N, total carbon / total nitrogen; DOC, dissolved organic carbon; NO_3^--N nitrate; NH_4^+-N, ammonium; DON, dissolved organic nitrogen; Ca, calcium; Mg, magnesium; Na, sodium; Fe, iron; Mn, manganese and Zn, zinc. Each physiochemical property is measured from its respective habitat. (**a**) root endosphere, (**b**) rhizosphere soil and (**c**) bulk soil.

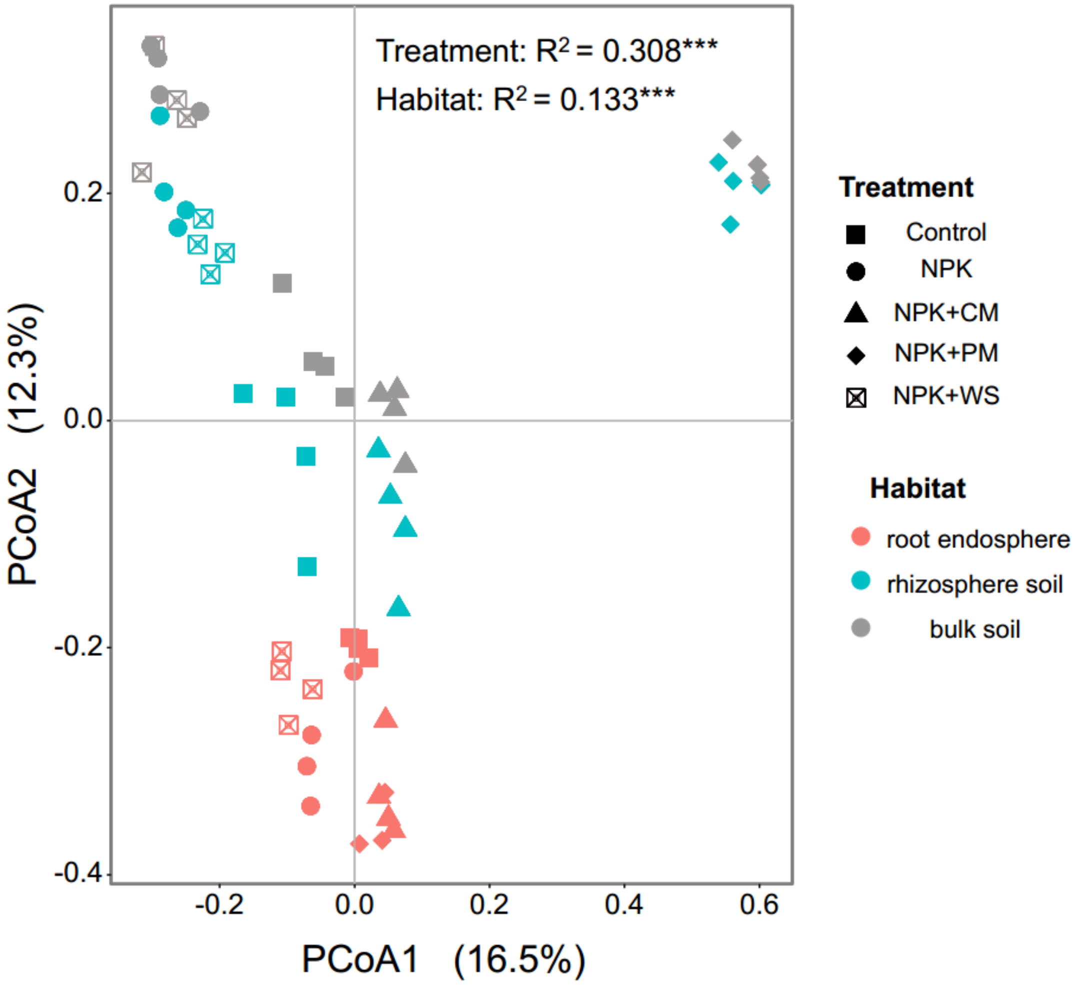

Fig. S2 Principal Coordinates Analysis (PCoA) of overall fungal communities based on Bray–Curtis distances with permutational analysis of variance (PERMANOVA) showing the effects of fertilization treatments and habitats on fungal communities.

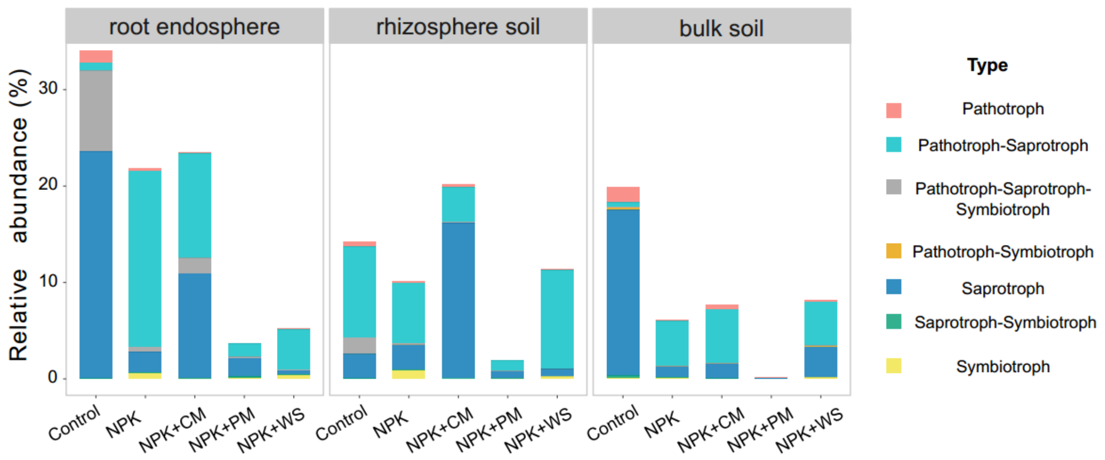

Fig. S3 Relative abundance of the fungal trophic type among different fertilization treatments in three habitats.

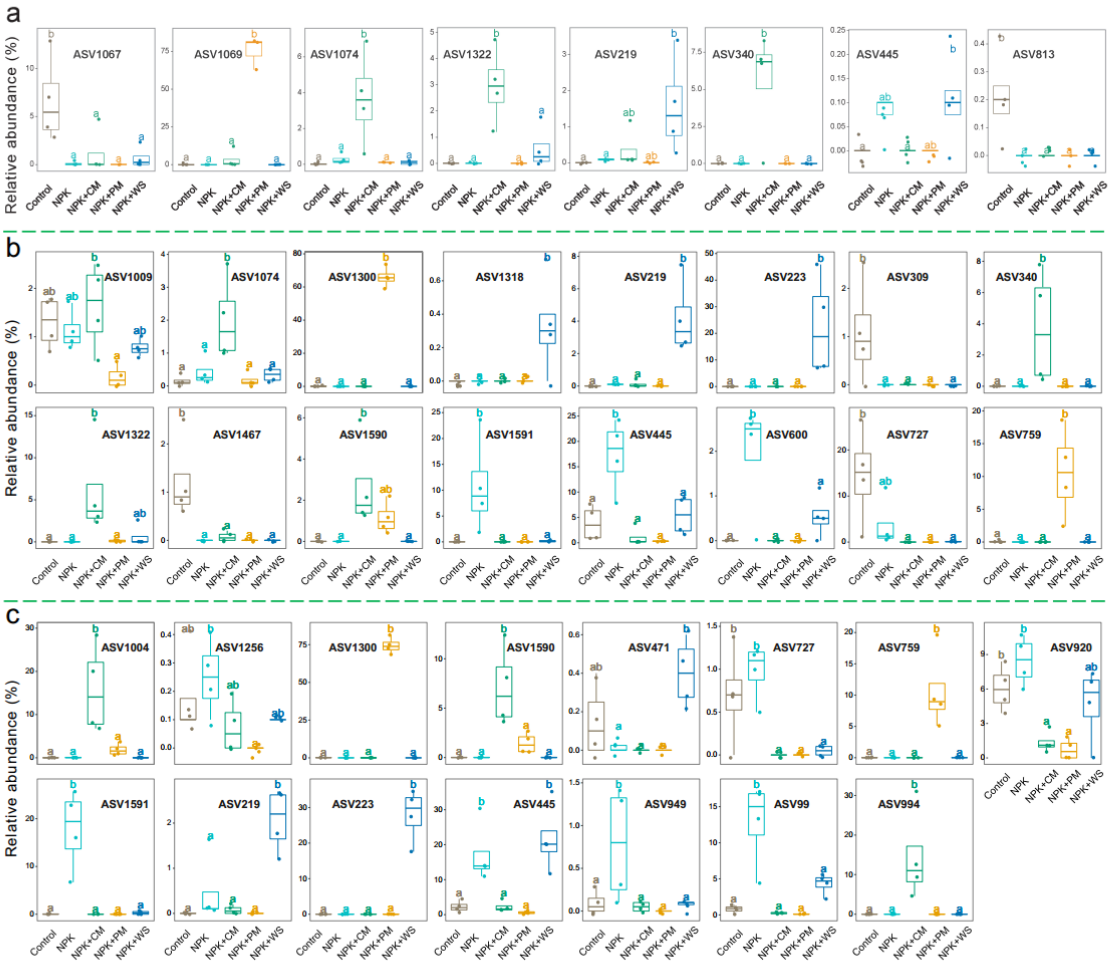

Fig. S4 Comparison on relative abundances of fungi at ASV level in three habitats (Only taxa had significant difference of top 50 abundant ASVs among treatments are showed). Values in the columns that do not share the same letter differ significantly (Duncan's test, $P < 0.05$). **(a)** root endosphere, **(b)** rhizosphere soil and **(c)** bulk soil.

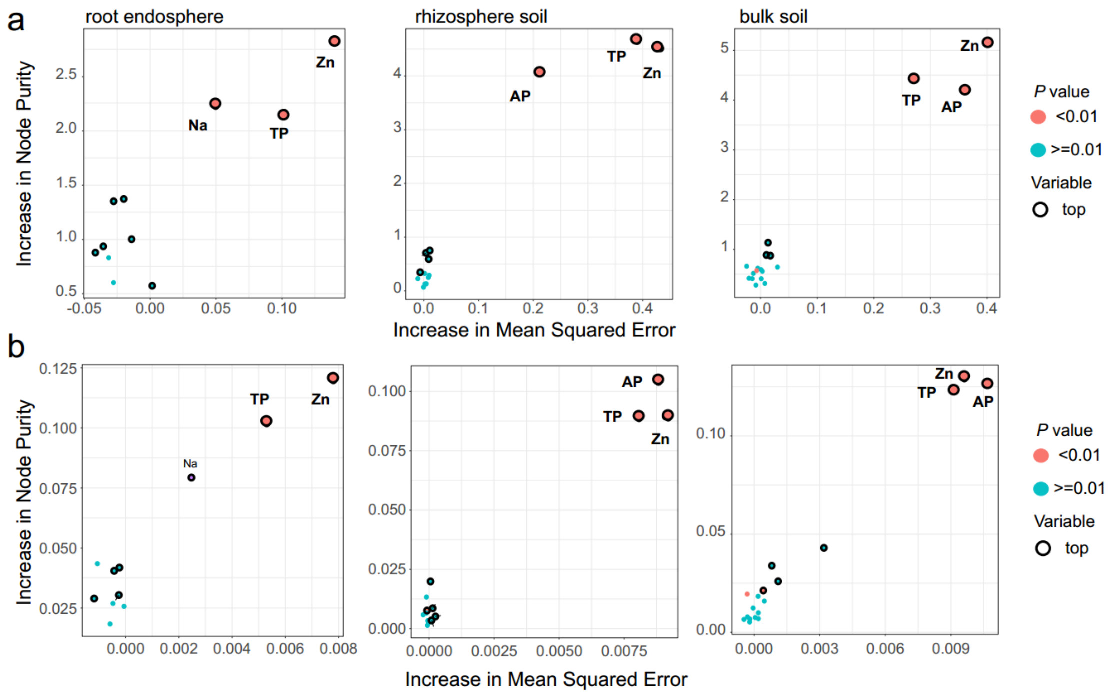

Fig. S5 Results of random forest analysis showing the relative contribution of various factors in determining the alpha diversity. Each physiochemical property is measured from its respective habitat. Abbreviation details showed in Fig S1. **(a)** Shannon and **(b)** Simpson index.

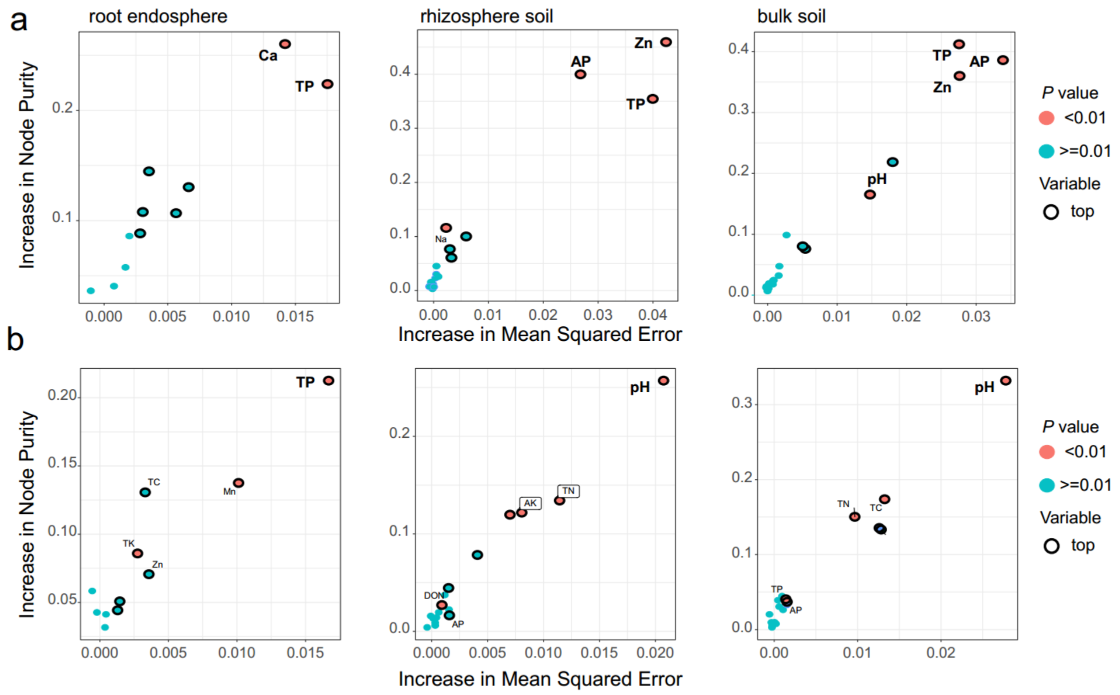

Fig. S6 Results of random forest analysis showing the relative contribution of various factors in determining the community structure . Each physiochemical property is measured from its respective habitat. Abbreviation details showed in Fig S1. **(a)** PCoA 1 and **(b)** PCoA 2.

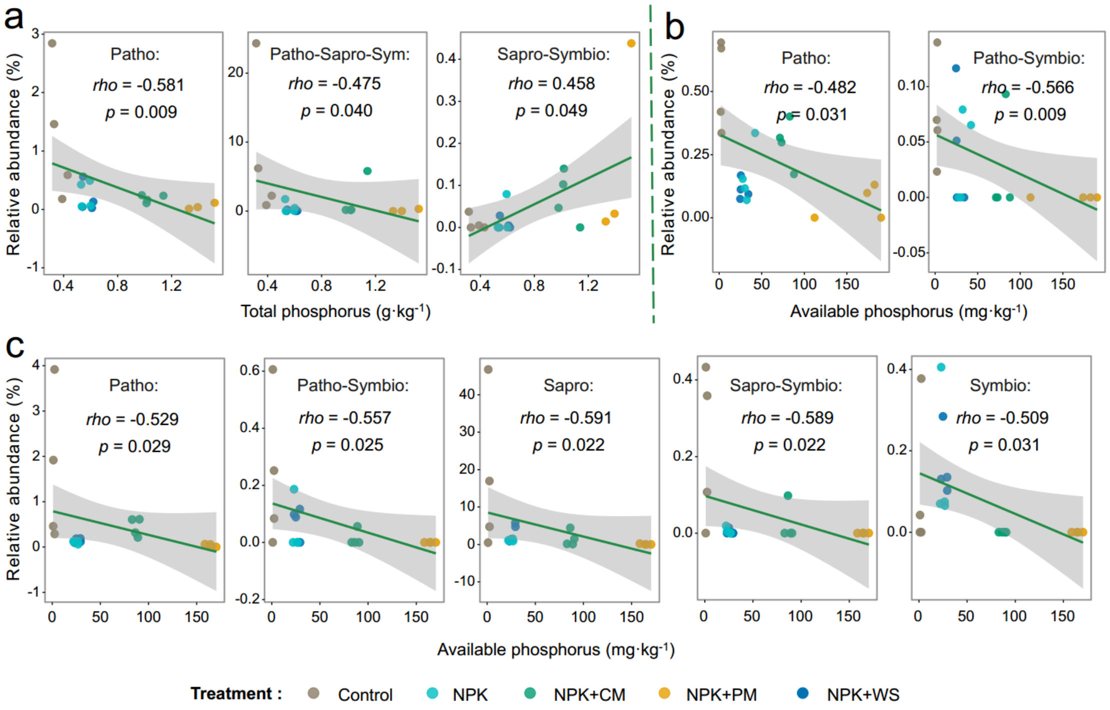

Fig. S7 Relationship between total phosphorus and the relative abundance of fungal trophic type. Data are fitted using linear regression and assessed by Spearman's rank correlation. Only significant relationships are showed. **(a)** root endosphere, **(b)** rhizosphere soil, and **(c)** bulk soil.

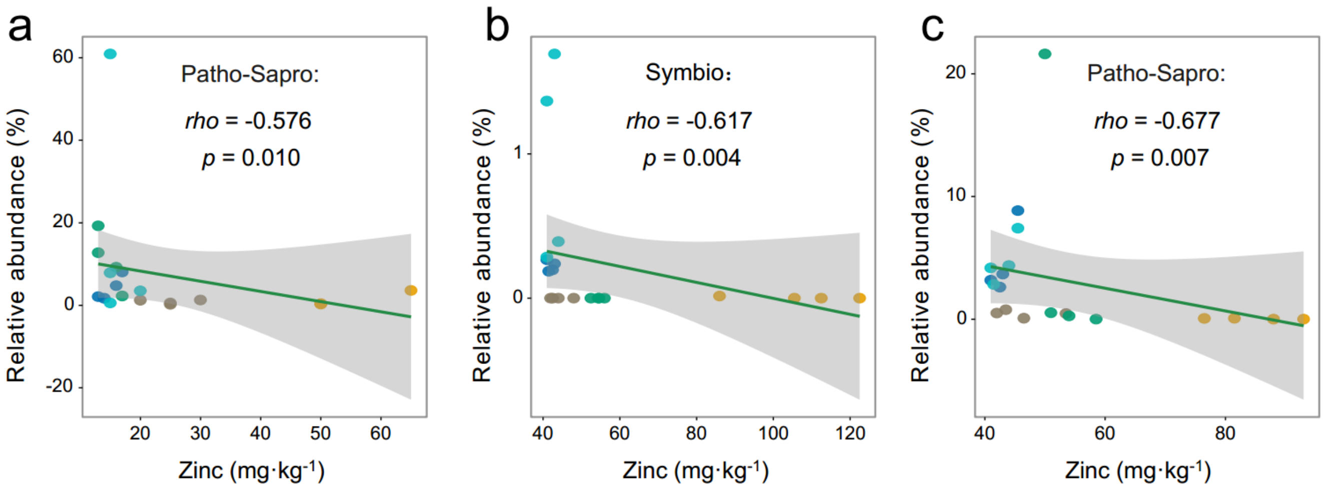

Fig. S8 Relationship between zinc and the relative abundance of fungal trophic type. Data are fitted using linear regression and assessed by Spearman's rank correlation. Only significant relationships are showed. **(a)** root endosphere, **(b)** rhizosphere soil, and **(c)** bulk soil.

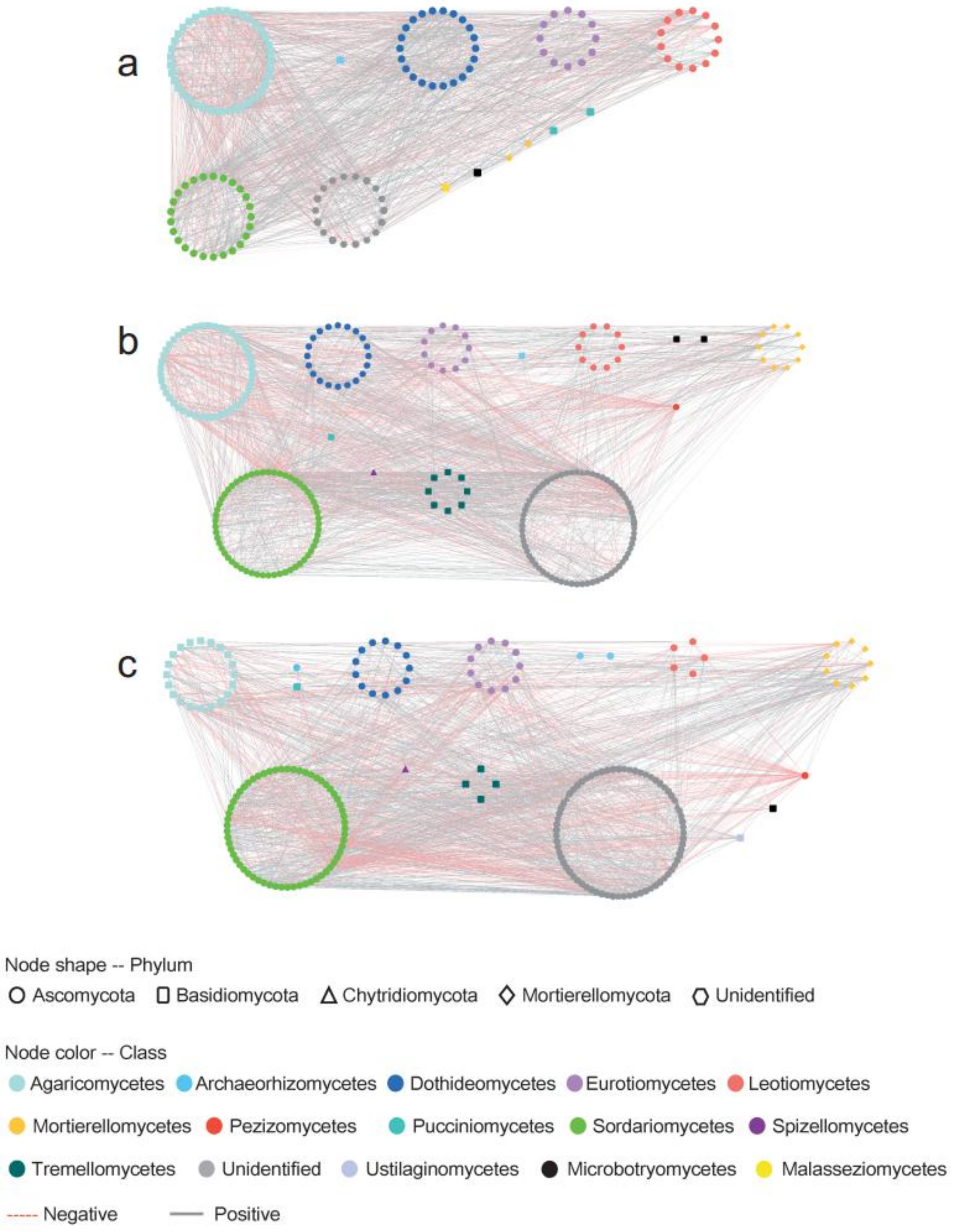

Fig. S9 Total co-occurrence network in **(a)** root endosphere, **(b)** rhizosphere soil and **(c)** bulk soil.

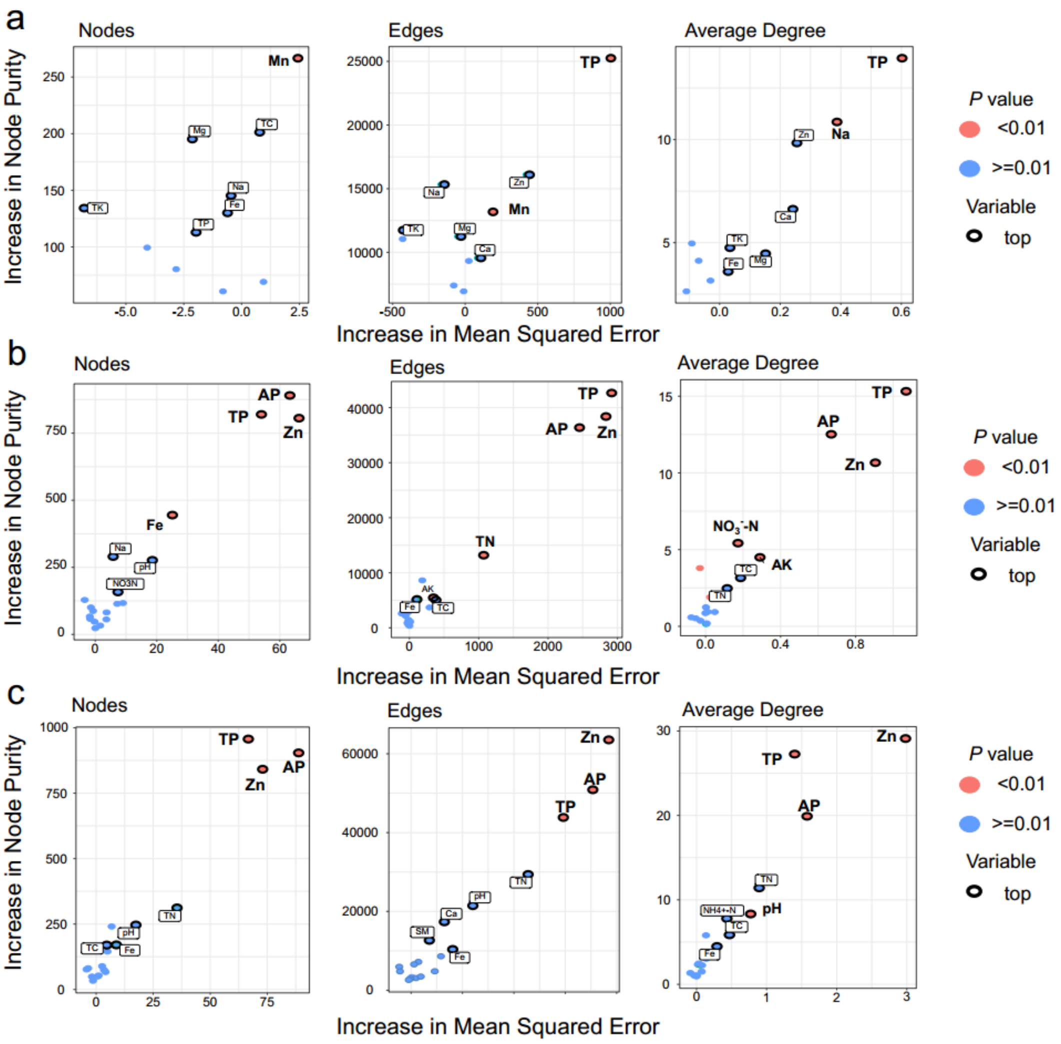

Fig. S10 Results of random forest analysis showing the relative contribution of various factors in determining the topological features of network. Each physiochemical property is measured from its respective habitat. Abbreviation details showed in Fig S1. **(a)** root endosphere, **(b)** rhizosphere soil and **(c)** bulk soil.
